# Inhaled nitric oxide and cognition in pediatric severe malaria: A randomized double-blind placebo controlled trial

**DOI:** 10.1371/journal.pone.0191550

**Published:** 2018-01-25

**Authors:** Paul Bangirana, Andrea L. Conroy, Robert O. Opoka, Michael T. Hawkes, Laura Hermann, Christopher Miller, Sophie Namasopo, W. Conrad Liles, Chandy C. John, Kevin C. Kain

**Affiliations:** 1 Department of Psychiatry, Makerere University College of Health Sciences, Kampala, Uganda; 2 Sandra A. Rotman Laboratories, Sandra Rotman Centre for Global Health, University Health Network-Toronto General Hospital, University of Toronto, Toronto, Canada; 3 Department of Paediatrics and Child Health, Makerere University College of Health Sciences, Kampala, Uganda; 4 Division of Pediatric Infectious Diseases, University of Alberta, Edmonton, Canada; 5 Department of Respiratory Medicine, Faculty of Medicine, University of British Columbia, Vancouver, Canada; 6 Department of Paediatrics, Jinja Regional Referral Hospital, Jinja, Uganda; 7 Department of Medicine, University of Washington, Seattle, Washington, United States of America; 8 Ryan White Center for Pediatric Infectious Disease and Global Health, Indiana University School of Medicine, Indianapolis, Indiana, United States of America; 9 Tropical Disease Unit, Division of Infectious Diseases, Department of Medicine, University of Toronto, Toronto, Canada; Utano National Hospital, JAPAN

## Abstract

**Background:**

Severe malaria is a leading cause of acquired neurodisability in Africa and is associated with reduced nitric oxide (NO) bioavailability. A neuroprotective role for inhaled NO has been reported in animal studies, and administration of inhaled NO in preterm neonates with respiratory distress syndrome is associated with a 47% reduced risk of cognitive impairment at two years of age.

**Methods:**

A randomized double-blind placebo-controlled trial of inhaled NO versus placebo as an adjunctive therapy for severe malaria was conducted in Uganda between 2011 and 2013. Children received study gas for a maximum 72 hours (inhaled NO, 80 parts per million; room air placebo). Neurocognitive testing was performed on children<5 years at 6 month follow-up. The neurocognitive outcomes assessed were overall cognition (a composite of fine motor, visual reception, receptive language, and expressive language), attention, associative memory, and the global executive composite. Main outcomes were attention, associative memory, and overall cognitive ability.

**Results:**

Sixty-one children receiving iNO and 59 children receiving placebo were evaluated. Forty-two children (35.0%) were impaired in at least one neurocognitive domain. By intention-to-treat analysis, there were no differences in unadjusted or unadjusted age-adjusted z-scores for overall cognition (β (95% CI): 0.26 (-0.19, 0.72), p = 0.260), attention (0.18 (-0.14, 0.51), p = 0.267), or memory (0.14 (-0.02, 0.30), p = 0.094) between groups by linear regression. Children receiving inhaled NO had a 64% reduced relative risk of fine motor impairment than children receiving placebo (relative risk, 95% CI: 0.36, 0.14–0.96) by log binomial regression following adjustment for anticonvulsant use.

**Conclusions:**

Severe malaria is associated with high rates of neurocognitive impairment. Treatment with inhaled NO was associated with reduced risk of fine motor impairment. These results need to be prospectively validated in a larger study powered to assess cognitive outcomes in order to evaluate whether strategies to increase bioavailable NO are neuroprotective in children with severe malaria.

**Trial registration:**

ClinicalTrials.gov Identifier: NCT01255215.

## Introduction

In sub-Saharan Africa, severe malaria leads to long-term neurocognitive deficits in 26% of survivors, making it a leading cause of acquired neurodisability [1], and the most common cause of disability-adjusted life-years (DALYs) in Africa following HIV/AIDS [2]. Deficits have been observed in attention, memory, speech and language with fewer studies reporting deficits in motor skills, visual spatial skills, executive function, somatosensory discrimination and learning [1, 3–12]. Neurologic deficits are typically observed in the first six months after the episode [13], with more subtle cognitive deficits persisting or emerging up to eight years post-illness [9].

Neurocognitive deficits after severe malaria have been associated with coma depth and duration, seizures, and neurological abnormalities at discharge [1, 5, 6, 8, 10, 11, 14]. Other factors associated with neurocognitive deficits include hypoglycemia, severe malnutrition, features of intracranial hypertension, and elevated cerebrospinal fluid tumor necrosis factor [1, 10, 14, 15].

Severe malaria is associated with decreased nitric oxide (NO) bioactivity as a result of NO production [16, 17]; inhibition of endothelial NO synthase (NOS) by endogenous inhibitors (e.g. asymmetric dimethyl arginine) [18]; reduced plasma L-arginine (a substrate for NOS) due to increased sequestration and consumption of intraerythrocytic arginine by the parasite; and NO quenching by cell-free hemoglobin [17, 19–22]. Administration of exogenous L-arginine in adults with severe malaria was associated with improved endothelial recovery [17], and delivery of inhaled NO (iNO) in experimental cerebral malaria was associated with prolonged survival and preserved blood brain barrier integrity [23]. Inhaled NO has an established safety record and is approved for use in neonates with hypoxic respiratory failure [24].

Based on the hypothesis that iNO is neuroprotective, cognitive outcomes were evaluated at six month follow-up as a secondary endpoint of a randomized controlled trial evaluating iNO as an adjunctive therapy for children with severe malaria [25].

## Methods

### Trial design

This was a prospective, parallel arm, randomized, double-blind placebo-controlled, clinical trial of iNO versus placebo among children with severe malaria treated with parenteral artesunate at Jinja Regional Referral Hospital in eastern Uganda where malaria accounts for over 30% of admissions. Children were eligible if they were aged 1–10 years with a positive three band malaria rapid diagnostic test (First Response Malaria Ag. (pLDH/HRP2) Combo Rapid Diagnostic Test, Premier Medical Corporation Limited, India), had features of severe malaria (repeated seizures, prostration, impaired consciousness, or respiratory distress), and were willing and able to complete follow-up [25]. Exclusion criteria included baseline methemoglobinemia (>2%), history of chronic illness, and severe malnutrition [25].

### Interventions

Simple randomization was performed using a computer-generated list created prior to study commencement [25]. Treatment assignment was recorded on paper and kept in sequentially numbered, sealed, opaque envelopes and stored in a locked cabinet accessible only to the un-blinded study team. Following patient stabilization and informed consent, the next envelope was drawn by an un-blinded investigator. All children were treated with intravenous artesunate according to the Ugandan Ministry of Health standard of care. Participants in the iNO group received the gas at a starting concentration of 80ppm administered continuously via non-rebreather facemask for a maximum period of 72 hours [25]. iNO was indistinguishable from placebo in appearance and delivery system (mask, tubing, vehicle air). An un-blinded monitoring team initiated and monitored study gas while blinded nurses and clinicians were out of the room. Flow-meters and monitoring devices were located in locked, opaque boxes, accessible only to the un-blinded study team.

### Outcomes

Neurocognitive functioning was a pre-specified secondary outcome of the study [26]. The primary outcome was the longitudinal recovery of angiopoietin-2, as previously published [25]. Overall cognitive ability was assessed by the Mullen Scales of Early Learning [27], attention was assessed by the Early Childhood Vigilance Test [28], associative memory was assessed by the Color Object Association Test [29], and executive function was assessed by the Behavior Rating Inventory of Executive Function [30]. The Mullen Scales, Color Object Association Test and Early Childhood Vigilance Test have been used in Ugandan children <5 years to study the long-term effect of severe malaria on cognition [6].

The Mullen Scales assess cognition and motor skills from birth to 68 months and is composed of five scales: i) gross motor, ii) fine motor, iii) visual reception, iv) receptive language, and v) expressive language. Cognitive ability is a summation of scores from four of five scales (excludes gross motor). Neurocognitive impairment is defined as a z-score ≤-2 in the following domains: fine motor, gross motor, visual reception, receptive language, and expressive language (from the Mullen Scales); associative memory (from the Color Object Association Test); and attention (from the Early Childhood Vigilance Test, ECVT). The summary score from all the items in the Behavior Rating Inventory of Executive Function is the Global Executive Composite, our measure of Executive Function [30]. A positive z-score in the Behavior Rating Inventory of Executive Function is associated with worse executive function, so impairment in the Global Executive Composite is defined as a z-score ≥2.

Cognitive testing was performed on children <5 years so the same set of neuropsychological tests could be used for the entire cohort. Based on the intensity of malaria transmission and pattern of illness in Jinja, the majority children presenting with severe malaria are<5 years old, and testing a limited sub-set of older children using a different set of tools would have limited value as tests cannot be directly compared when different assessment tools are used. Each child was assessed by a single neurocognitive tester blinded to treatment group and testing was performed according to the test manuals. For quality control assessment, every five ECVT videos were re-coded by the supervisor to ensure scores were within 10 seconds of each other. Once 30 videos were coded without problem, the coder was deemed proficient and coded videos independently.

### Statistical analysis

Inclusion of 180 children with severe malaria was needed to show with 80% power and 95% confidence a 50% difference in the rate of change in Ang-2 over hospitalization [25]. Age adjusted z-scores were generated for each of the outcomes as previously described [1]. Age adjusted z-scores for the neurocognitive outcomes were compared between the groups using linear regression adjusting for co-variates with a p<0.200 by bivariate analysis. Log binomial regression was used to estimate the relative risk between treatment arm and impairment across neurocognitive domains adjusting for anti-convulsant use. To compare z-scores with recovery times we used Spearman’s non-parametric correlation. Statistical analyses were done with IBM® SPSS statistics for Windows, version 20.0 (IBM Corp., Armonk, N.Y., USA) and Stata version 14.0 (StataCorp. 2015. Stata Statistical Software: Release 14. College Station, TX: StataCorp LP). An alpha of less than 0.05 was considered statistically significant. To adjust for multiple comparisons we used the Bonferroni correction and an adjusted alpha of 0.006 is required for significance based on 9 neurocognitive domains assessed (attention, memory, executive function, cognition, and five cognitive sub-scales).

### Ethical considerations

Written informed consent was obtained from guardians of study participants. Ethical approval was granted by the Institutional Review Boards at Makerere University School of Medicine, University Health Network, Toronto, and the Uganda National Council of Science and Technology. The trial is registered (ClinicalTrials.gov Identifier: NCT01255215).

## Results

**Fig 1:**
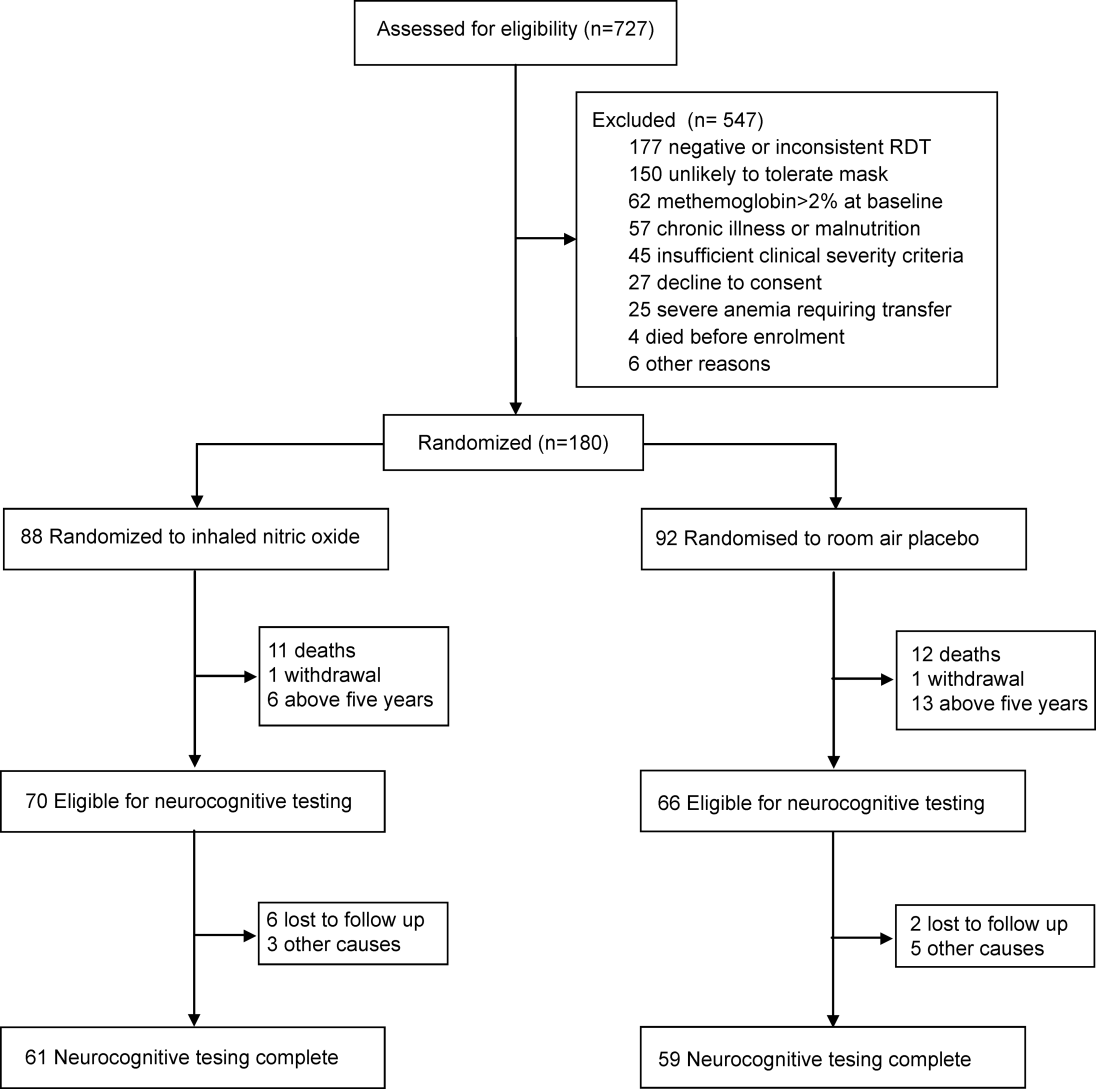
Flowchart of study population.

Participants were recruited between July 2011 and June 2013 and followed until January 2014 according to the study protocol. Nineteen of the original 180 children were not eligible for neurocognitive follow up as they were greater than five years of age. The flow of study participants is shown in **Fig 1**. The percentage of children completing neurocognitive testing at 6 months was comparable between groups (iNO, n = 61/70, 87%; placebo, n = 59/66, 89%). The time to neurocognitive evaluation was 26.5 weeks in the iNO group versus 26.3 weeks in the placebo group (p = 0.77).

There was no difference in age, sex, weight-for-age z-score, socioeconomic status, parental education, and child’s education between arms (**Table 1**). The number of presenting features of severe malaria was similar between trial arms (**Table 1**). Children randomized to iNO were more likely to receive an anticonvulsant during hospitalization (diazepam and/or phenobarbitone, p = 0.013). The mean duration of time on study gas was comparable between groups with children receiving iNO for 61.4 hours versus hours 63.0 in the placebo group (p = 0.496). At discharge, surviving children had a neurologic exam; 8 (9.8%) children in the placebo group and 5 (6.2%) children in the iNO group had objective neurologic abnormalities (p = 0.40). Abnormalities included inability to sit (2), unilateral weakness (3), abnormal tone (2), extra-ocular muscle palsy (2), visual defects (3), seizure disorder (2), and inability to speak (2). Computed tomography of the head was performed in 4 patients and showed global atrophy in 2 cases, suggestive of antecedent insult. Full recovery to baseline neurologic function by day 14 was apparent in only one patient.

**Table 1:** Demographic characteristics of follow-up cohort.

	iNO (n = 61)	Placebo (n = 59)	P value
**Admission Characteristics**			
Age (years)	2.1 (1.4, 2.7)	2.1 (1.4, 3.0)	0.545
Sex (n, % female)	21 (34.4)	29 (49.2)	0.102
Weight for age z-score	-0.6 (-1.7, 0)	-1.0 (-1.8, -0.4)	0.122
Height, cm	78 (72, 87)	80 (72, 87)	0.745
History of fever, days	3 (2, 5)	3 (2, 4)	0.495
Respiratory rate	48 (40, 62)	50 (39, 60)	0.875
Coma on admission (BCS<3) (n, %)	38 (62.3)	31 (52.5)	0.280
Hyperlactatemia on admission (lactate>5mmol/L) (n, %)	14 (23.7)	23 (39.0)	0.074
Severe anemia on admission (n, %)	37 (60.7)	40 (67.8)	0.415
Hypoglycemia (glucose <2.2mmol/L) (n, %)	0 (0.0)	2 (3.4)	0.240
Hemoglobin, g/dL	5.4 (3.6, 6.7)	4.5 (3.1, 6.5)	0.295
Lactate, mmol/L	3.2 (2.1, 4.6)	3.9 (2.3, 7.9)	0.093
Glucose, mmol/L	6.7 (5.6, 8.5)	6.9 (5.6, 8.7)	0.688
Creatinine, umol/L	29 (21, 36)	32 (25, 42)	0.051
Number of severe malaria criteria^a^	4 (3, 6)	5 (3, 5)	0.619
Pretreatment with antibiotics (n, %)	31 (54.4)	26 (45.6)	0.349
Pretreatment with antimalarials (n, %)	36 (60.0)	38 (64.4)	0.620
**Household Characteristics**			
Socioeconomic status score	9 (8, 11)	9 (7, 10)	0.151
Maternal education (n, %)			0.357
Primary 6 or lower	24 (40.7)	22 (39.3)	
Primary 7	13 (22.0)	10 (17.9)	
Secondary or higher	18 (30.5)	17 (30.4)	
Not known	4 (6.8)	7 (125)	
Paternal education (n, %)			0.931
Primary 6 or lower	12 (20.0)	12 (21.1)	
Primary 7	14 (23.3)	12 (21.1)	
Secondary or higher	33 (55.0)	31 (54.4)	
Not known	1 (1.7)	2 (3.5)	
Child’s education, pre-school (n, %)	4 (6.7)	2 (3.5)	0.680

Data presented as median (IQR)

^a^ Coma, respiratory distress, convulsions, prostration, hypoglycemia, hyperlactatemia, severe anemia, shock, acute kidney injury, jaundice.

Analysed using Wilcoxon rank sum, or Pearson Chi-Square or Fisher’s exact (as appropriate).

### Factors associated with neurologic abnormality and neurocognitive outcome

The proportion of children with a neurologic abnormality at discharge and neurocognitive impairment at 6 months follow-up were 8.0% and 35.0% respectively. Children with neurologic abnormality at discharge were more likely to be comatose (p = 0.007), had a lower BCS at admission (p<0.0001), took longer to localize pain (p = 0.003) and regain consciousness (p<0.0001) than children with a normal neurologic exam at discharge. Neurologic abnormality at discharge was associated with impairments in fine and gross motor skills (p<0.001 for both), receptive language (p = 0.001), expressive language (p = 0.005), overall cognition (p = 0.008), and executive function (p = 0.001). Neurologic abnormality at discharge was associated with a greater number of cognitive domains impaired (p<0.001, Chi-Square test-for-trend).

As this study enrolled children with multiple manifestations of severe malaria, we investigated the nature of neurologic abnormality and cognitive impairment in children with and without coma. Children with coma on admission had lower mean z-scores for gross and fine motor functioning (all p<0.05) compared to children with a BCS>2. There were no differences in the mean z-scores in other domains assessed or in rates of neurocognitive impairment according to coma status on admission (p>0.05 for all domains assessed). Treatment with anticonvulsants was not associated with neurologic deficit at discharge or the presence of any cognitive deficit at 6 months follow up. Administration of anticonvulsants was associated with worse z scores in expressive language (mean difference (95% CI, -0.25 (-0.42 to -0.08), p = 0.0498), but was not associated with differences in the other cognitive domains assessed.

We investigated the association between clinical recovery times and cognitive outcomes at six months. Children receiving iNO had a significantly longer duration of hospitalization and took longer to achieve a BCS of 5 than children in the placebo arm (median (IQR), p-value: duration of hospitalization, iNO, 79 hours (62–91), placebo, 63 hours (59, 85), p = 0.033; time to reach BCS 5, iNO, 15.3 hours (9.3, 29.5), placebo, 12.0 (6.0, 22.7), p = 0.051) (**Table 2**). There was no difference in other recovery times between treatment arm.

**Table 2:** Complications, recovery times, and neurocognitive outcome by treatment arm.

	iNO (n = 61)	Placebo (n = 59)	P
**Cotreatments**			
Blood transfusion	47 (77.1)	45 (76.3)	0.920
Antibiotic			
Ceftriaxone	57 (95.0)	51 (89.5)	0.262
Metronidazole	4 (7.0)	3 (5.9)	0.811
Other	5 (8.2)	7 (12.3)	0.463
Anticonvulsant			
Diazepam	26 (45.6)	15 (30.6)	0.114
Phenobarbitone	20 (33.9)	12 (20.3)	0.098
Diazepam or Phenobarbitone	35 (58.3)	21 (35.6)	0.013
Antipyretic			
Paracetamol	46 (76.7)	49 (83.1)	0.386
Diclofenac	6 (10.9)	5 (10.4)	0.936
**Complications**			
Convulsions persisting >6h after admission	12 (19.7)	10 (16.9)	0.815
Develop severe anemia^a^	9 (34.6)	3 (16.7)	0.303
Develop hemoglobinuria^a^	0 (0.0)	3 (5.9)	0.118
Treatment discontinued for acute kidney injury^b^	3 (4.9)	0 (0.0)	0.244
Persistent acidosis at 12 hours (lactate>5mmol/L)	8 (15.1)	4 (7.5)	0.359
**Recovery Times**			
Time to first feed, hours	19.0 (12.1, 37.2)	13.6 (8.5, 26.2)	0.073
Time to fever resolution, hours	8.0 (0, 32.0)	9.0 (0, 28.5)	0.920
Time to localize pain, hours	8.2 (0, 15.9)	5.7 (0, 12.3)	0.172
Time to BCS 5, hours	15.3 (9.3, 29.5)	12.0 (6.0, 22.7)	0.051
Time to first sit, hours	34.3 (17.5, 60.5)	32.0 (15.3, 62.2)	0.070
Duration of hospitalization	79.0 (62.0, 90.5)	63.0 (59.0, 84.5)	0.033
**Neurocognitive Outcomes**			
Neurologic abnormality on discharge	4 (6.6)	5 (8.5)	0.741

Continuous data analyzed using Wilcoxon rank sum test otherwise indicated; categorical variables analyzed using Pearson’s Chi-Square test or Fisher’s exact, as appropriate.

^**a**^ Assessed only in children without these disorders on admission.

^b^ Acute kidney injury was defined as follows: Serum creatinine >1.5 × upper limit of normal (children age 1–2) or >2.0 × upper limit of normal (age 2–10) AND an abrupt (within 48 h) reduction in kidney function: (1) an absolute increase in serum creatinine of ≥26.4 μmol/l; or (2) a percentage increase in serum creatinine of ≥50%.

In the placebo arm, prolonged clinical recovery times were associated with worse cognitive functioning. Increased time to sit unsupported was associated with lower attention scores (rho, p-value: -0.33, p = 0.02) and worse gross motor functioning (-0.36, p = 0.006). Time to localize pain was inversely associated with overall cognitive score (-0.32, p = 0.01), gross motor functioning (-0.27, p = 0.04), receptive language (-0.26, p = 0.04), and expressive language (-0.26, p = 0.05). Increased duration of hospitalization was associated with worse gross motor functioning (-0.27, p = 0.04). In children receiving iNO, there was an inverse association between the duration of hospitalization and fine motor functioning (-0.34, p = 0.007). Complications and clinical recovery times between groups are shown in **Table 2**.

### Effect of iNO versus placebo on neurocognitive outcome

By intention-to-treat analysis iNO was not associated with improved overall cognition, memory, or attention (p>0.05) in age-adjusted z scores (**Table 3, Table 4**). In a linear regression analysis adjusting for sex, socioeconomic status, venous lactate, creatinine and anticonvulsant use between groups, there was no difference in age-adjusted z scores by treatment arm (p>0.05, **Table 4**). However, treatment with iNO was associated with less fine motor impairment than children receiving placebo (iNO, n = 5 (8.2%); placebo, n = 13 (22%), p = 0.03), corresponding to a 64% reduced relative risk of fine motor impairment when receiving iNO (RR, 95% CI: 0.36, 0.14–0.96) adjusting for anticonvulsant use (p = 0.04, **Table 3**). Further, children in the placebo arm were more likely to have multiple domains impaired with 25.4% of children in placebo arm having two or more domains impaired compared to 11.5% in the nitric oxide group (p = 0.048 by Pearson’s Chi Square). Inhaled nitric oxide was associated with a 58% reduced relative risk of having multiple domains impaired (RR, 95% CI: 0.42, 0.18–0.97, p = 0.04, **Table 3**) following adjustment for anticonvulsant use. The proportion of children impaired in other domains assessed was comparable between arms (**Table 3**).

**Table 3:** Differences in mean age-adjusted z scores and cognitive impairment by study arm.

	iNO (n = 61)	Placebo (n = 59)	Effect Size	P
**Age-adjusted z-scores**			**Mean difference (95% CI)**	
**Attention (ECVT)**	**0.70 (0.86)**	**0.52 (0.91)**	**-0.18 (- 0.51 to 0.14)**	**0.268**
**Associative Memory (COAT)**	**-0.04 (0.41)**	**-0.14 (0.49)**	**-0.14 (-0.30 to 0.02)**	**0.095**
Global Executive Composite (BRIEF)*	0.59 (1.00)	0.69 (1.41)	0.10 (-0.34 to 0.55)	0.653
**Overall Cognitive Ability (Mullen)**	**-0.61 (1.09)**	**-0.87 (1.41)**	**-0.26 (-0.72 to 0.20)**	**0.262**
Fine Motor	-0.58 (1.23)	-0.99 (1.68)	-0.41 (-0.94 to 0.12)	0.131
Gross Motor	-0.08 (1.76)	-0.14 (2.00)	-0.06 (-0.75 to 0.62)	0.852
Visual Reception	-0.91 (1.35)	-1.10 (1.56)	-0.19 (-0.72 to 0.33)	0.470
Receptive Language	-0.70 (1.09)	-0.95 (1.51)	-0.25 (-0.73 to 0.22)	0.300
Expressive Language	-0.03 (0.78)	-0.10 (0.81)	-0.06 (-0.35 to 0.22)	0.665
**Impairment**			**Relative Risk (95% CI)**	**P**
**Attention (ECVT)**	**0 (0.0%)**	**1 (1.8%)**	**---**	**—**
**Associative Memory (COAT)**	**0 (0.0%)**	**0 (0.0%)**	**---**	**---**
Global Executive Composite (BRIEF)*	5 (8.3%)	8 (13.6%)	0.47 (0.17, 1.36)	0.165
**Overall Cognitive Ability (Mullen)**	**5 (8.2%)**	**8 (13.6%)**	**0.64 (0.22, 1.91)**	**0.428**
Fine Motor	5 (8.2%)	13 (22.0%)	0.36 (0.14, 0.96)	0.042
Gross Motor	3 (4.9%)	5 (6.4%)	0.76 (0.17, 3.35)	0.712
Visual Reception	15 (24.6%)	14 (23.7%)	0.99 (0.52, 1.90)	0.985
Receptive Language	4 (6.6%)	10 (16.9%)	0.39 (0.13, 1.21)	0.103
Expressive Language	1 (1.6%)	1 (1.7%)	0.96 (0.06, 16.10)	0.976
Any domain impaired	18 (29.5%)	24 (40.7%)	0.69 (0.42, 1.14)	0.150
Multiple domains impaired	7 (11.5%)	15 (25.4%)	0.42 (0.18, 0.97)	0.042

Main outcomes are in bold.

Data are presented as mean (SD) or n (%) with analysis by Student’s t-test or a log binomial model adjusting for anticonvulsant use

Impairment is defined as less than -2SD in age-adjusted z-scores for the early learning composite (encompassing fine/gross motor, visual reception, receptive/expressive language), attention, and associative memory, or greater than 2SD for the global executive function.

* A positive z-score in the Global Executive Composite (BRIEF) is associated with worse executive function.

**Table 4:** Regression models of unadjusted and adjusted age-adjusted z scores by study arm.

	Unadjusted Model		Adjusted Model^1^	
	Beta (95% CI)	P	Beta (95% CI)	P
**Attention (ECVT)**	0.18 (-0.14, 0.51)	0.267	0.18 (-0.18, 0.54)	0.333
**Associative Memory (COAT)**	0.14 (-0.02, 0.30)	0.094	0.12 (-0.07, 0.30)	0.193
Global Executive Composite (BRIEF)*	-0.10 (-0.55, 0.34)	0.652	-0.28 (-0.74, 0.18)	0.228
**Overall Cognitive Ability (Mullen)**	0.26 (-0.19, 0.72)	0.260	0.33 (-0.20, 0.85)	0.219
Fine Motor	0.41 (-0.12, 0.94)	0.129	0.50 (-0.11, 1.11)	0.110
Gross Motor	0.06 (-0.62, 0.74)	0.852	0.37 (-0.39, 1.14)	0.336
Visual Reception	0.19 (-0.33, 0.72)	0.469	0.26 (-0.35, 0.86)	0.405
Receptive Language	0.25 (-0.22, 0.73)	0.297	0.21 (-0.34, 0.76)	0.448
Expressive Language	0.06 (-0.22, 0.35)	0.665	0.14 (-0.18, 0.46)	0.391

Main outcomes are in bold.

^1^ Data analyzed by linear regression with age-adjusted z scores as dependent variables and sex, socioeconomic status, venous lactate, anticonvulsant use, and creatinine as independent variables.

* A positive z-score in the Global Executive Composite (BRIEF) is associated with worse executive function.

## Discussion

This is the first randomized controlled trial to investigate neurocognitive outcomes in children receiving an adjunctive therapy for severe malaria. There proportion of children with neurocognitive impairment at follow-up was high with 35% of children tested demonstrating cognitive impairment in at least one domain. The impact of iNO on neurologic and neurocognitive outcomes was analyzed by intention-to-treat analysis. There were no differences in age adjusted z-scores between children receiving iNO vs. placebo looking at individual neurocognitive domains (e.g. attention, fine/gross motor skills, expressive/receptive language, associative memory) or composite measures (e.g. overall cognition, executive function), but children receiving iNO were less likely to have fine motor impairment (p = 0.04), although the effect was not significant following adjustment for multiple comparisons. Collectively, these data show there is a high risk of neurocognitive impairment following severe malaria even when treated with intravenous artesunate and highlight the importance of including cognitive testing when evaluating therapies for severe malaria.

A neuroprotective role for iNO has been reported in animal studies [31–33], and administration of iNO in preterm neonates with respiratory distress syndrome is associated with a 47% reduced risk of cognitive impairment at two years of age [24]. In this study we observed reduced fine motor impairment in children randomized to iNO supporting the hypothesis that iNO is neuroprotective in severe malaria; however, as this study was not powered for neurocognitive outcomes, it is likely we were underpowered to detect differences across multiple domains. Further, in this study a heterogeneous group of children with severe malaria with and without neurologic manifestations were included. The majority of studies assessing cognitive impairments following severe malaria have focused on children with neurologic signs (e.g. coma, multiple seizures, and decreased consciousness). Unexpectedly, we observed only modest differences in cognitive performance in children who presented with coma versus those without; suggesting cognitive impairment is common even in children without clear neurologic manifestations at presentation. These data are consistent with a recent report documenting cognitive impairment following severe malarial anemia [6].

In the original trial report, there were trends of lower mortality and prolonged coma duration times in children randomized to iNO, and children receiving iNO were more likely to receive anticonvulsants [25]. As the study was not powered to detect differences in mortality, it raised questions of whether iNO led to better survival but was associated with increased coma duration, neurologic sequelae and cognitive impairment. The data presented herein do not support this. While we observed increased duration of hospitalization and prolonged recovery to BCS 5 in children receiving iNO in the subset of children tested at 6 months follow up, children receiving iNO had less fine motor impairment and were less likely to have multiple domains impaired than children receiving placebo. Although the neurocognitive difference was not significant following adjustment for multiple comparisons, it is important to note that the study was not powered for neurocognitive outcomes.

Limitations of this study include a relatively short follow-up period with a single cognitive assessment. A longer follow-up period would permit an evaluation of children as they start school and have greater cognitive demands. This is important as cognitive deficits have been shown to persist over at least a year of follow up [6]. The sample size for this study was calculated based on estimated longitudinal changes in angiopoietin-2 over hospitalization. We were likely underpowered to detect more subtle cognitive differences between trial arms, particularly as the population consists of a heterogeneous group of children with different manifestations of severe malaria. As cognitive assessment tools are validated within specific age ranges we were limited to evaluating neurocognitive function to children <5 years of age given the mean age of children presenting with severe malaria in our population.

Strengths of this study include the randomized study design where all clinicians, parents, and neuropsychological testers were blinded to the treatment group. Further, a comprehensive set of neurocognitive assessment tools that have been validated for use in Ugandan children were used to assess neurocognitive recovery. Importantly, in this study we show neurocognitive impairment was common in a heterogeneous group of children with severe malaria treated with artesunate. Additional studies to validate these studies are needed and should consider including a longer period of follow up and larger sample size to delineate the risk of cognitive impairment across common manifestations of severe malaria in children.

## Conclusion

Our data suggest iNO may be neuroprotective in Ugandan children with severe malaria with improvements in fine motor functioning in children receiving iNO. Although these data require validation, this study is the first to suggest a beneficial long-term impact associated with an adjunctive therapy in severe malaria. Cognitive impairment was common among all children, suggesting additional studies are required to define the extent and the mechanisms that underlie cognitive impairment in severe malaria. As more data become available on the frequency and breadth of cognitive impairment across the spectrum of severe malaria, better estimates on the long term impact of severe malaria will emerge. Given the impact of malaria on DALYs in Africa, it is a public health imperative that treatments be identified to limit neurodisability (e.g. adjunctive neuroprotective therapies), and promote neurocognitive recovery. The high risk of cognitive impairment in children treated with artesunate strongly support inclusion of neurocognitive assessment in trials assessing adjunctive therapies in children with severe malaria.

## Supporting information

S1 File Data. (XLS)

S2 File CONSORT checklist. (DOC)

S3 File IRB approved trial protocol. (PDF)
