# Supplementary material for: Inhaled nitric oxide and cognition in pediatric severe malaria: A randomized double-blind placebo controlled trial
Source: PLoS One. 2018 Jan 25;13(1):e0191550. doi: 10.1371/journal.pone.0191550 (PMC5784958; doi:10.1371/journal.pone.0191550)
Supplement: S3 File — (PDF) [file pone.0191550.s003.pdf]

Inhaled Nitric Oxide for the Adjunctive Therapy of Severe Malaria:

a Randomized Controlled Trial

Principal investigator (Canada): Dr. Kevin Kain, *MD, FRCPC,* Professor of Medicine, Canada Research Chair in Molecular Parasitology, Director of Global Health, McLaughlin Center for Molecular Medicine, University of Toronto, Director, Sandra A. Rotman Laboratories, McLaughlin-Rotman Centre for Global Health, Director, Center for Travel and Tropical Medicine, Toronto General Hospital

Principal investigator (Uganda): Dr. Robert Opika Opoka, *MB Ch.B., M.Med., MPH.,*Department of Pediatrics & Child Health, Mulago Hospital

Co-Investigators:

Dr. Abner Tagoola, *MBChB, MMED, MPH*, Jinja Regional Referral Hospital

Dr. Sophie Namasopo, *MBChB, MMED,* Jinja Regional Referral Hospital

Dr. Chandy C. John, *M.D., M.S.*, Department of Pediatrics, University of Minnesota.

Dr. W. Conrad Liles, *MD, PhD,* Dept of Medicine, University of Toronto

Dr. Michael Hawkes, *MD, FRCPC*, Institute of Medical Sciences, Univ of Toronto

Dr. Christopher Miller, *PhD*, University of British Columbia

Version 8: 21 Oct, 2011

TABLE OF CONTENTS

Protocol Summary 4
1 General information 6
1.1 Title: Inhaled Nitric Oxide for the Adjunctive Therapy of Severe Malaria: a Randomized Controlled Trial 6
1.2 Sponsors 6
1.3 Name and title of person authorized to sign the protocol and the protocol amendments for sponsor: 6
1.4 Medical expert for the trial 6
1.5 Investigators 7
1.6 Physician responsible for all trial-site related medical decisions 8
1.7 Laboratories and other institutions involved in the trial 8
2 Background information 8
2.1 Description of Investigational Product 9
2.2 Inhaled nitric oxide in clinical practice and clinical trials 21
2.3 Potential risks and benefits 25
2.4 Justification of route of administration, dosage, dosage regimen, and treatment period 26
2.5 Statement of compliance 26
2.6 Description of the population to be studied 27
3 Trial objectives 27
4 Trial design 28
4.1 Endpoints 28
4.2 Trial design 31
4.3 Description of measures taken to minimize bias 33
(a) Randomization 33
(b) Blinding 33
4.4 Investigational treatment 34
4.5 Expected duration of subject participation 34
4.6 Description of stopping rules/discontinuation criteria 35
4.7 Accountability procedures, including placebo and comparators 37
4.8 Maintenance of the trial treatment randomization codes and procedures for breaking codes 37
4.9 Data to be included on the case report forms (CRF) 38
5 Selection and withdrawal of subjects 43
5.1 Inclusion criteria 43
5.2 Exclusion criteria 44
5.3 Subject withdrawal criteria (terminating trial treatment) 45
(a) When and how to withdraw subjects from the trial 45
(b) The type and timing of data to be collected for withdrawn subjects: 46
(c) Whether and how subjects are to be replaced: 46
(d) The follow-up for subjects withdrawn from trial treatment 47
6 Treatment of subjects 47

6.1 Description of intervention 47
6.2 Co-administered treatments during the trial 49
6.3 Procedures for monitoring subject compliance 49
7 Assessment of efficacy 50
7.1 Specification of efficacy parameters 50
7.2 Methods and timing for assessing, recording, and analyzing efficacy parameters 52
8 Assessment of safety 53
8.1 Specification of safety parameters 53
8.2 The methods and timing for assessing, recording, and analysing safety parameters 56
8.3 Procedures for eliciting reports of and for recording and reporting adverse event and intercurrent illnesses 58
8.4 The type and duration of the follow-up of subjects after adverse events 59
9 Statistics 59
9.1 Description of statistical methods to be employed 59
9.2 Number of subjects planned to be enrolled 62
9.3 Level of significance to be used 64
9.4 Criteria for termination of the trial 64
9.5 Procedure for accounting for missing, unused, and spurious data 64
9.6 Procedures for reporting deviations from the original statistical plan 65
9.7 Selection of subjects to be included in analyses 65
10 Direct access to source data and documents 66
11 Ethics 66
12 Quality control and quality assurance 72
13 Data handling and recordkeeping 76
14 Publication policy 78
References 81
Appendix 1. Statistical thresholds for monitoring mortality 86

PROTOCOL SUMMARY

Title: **Inhaled Nitric Oxide as Adjunctive Therapy for Malaria: a Randomized Controlled Trial**

Background: Severe malaria remains a major cause of global morbidity and mortality. While the use of artemisinin-based antimalarial therapy has improved outcomes in severe malaria, the mortality rate remains high. Adjunctive therapies that target the underlying pathophysiology of severe malaria may further reduce morbidity and mortality. Endothelial activation plays a central role in the pathogenesis of severe malaria, of which the angiogenic factors angiopoietin-1 (Ang-1) and angiopoietin-2 (Ang-2) have recently been shown to function as key regulators. Nitric oxide (NO) is a major inhibitor of Ang-2 release from endothelium and has been shown to decrease endothelial inflammation and reduce the adhesion of parasitized erythrocytes. Low-flow inhaled nitric oxide gas (iNO) is a US FDA-approved treatment for hypoxic respiratory failure in neonates. Based on compelling data on the efficacy of iNO in experimental cerebral malaria in animal models, coupled with the documented safety of iNO in clinical practice and trials for other diseases, we propose a randomized clinical trial of iNO for the adjunctive treatment of severe malaria in Ugandan children.

Population: 180 children 1-10 years old admitted to hospital with moderately severe and severe malaria; 90 treated with iNO (80ppm) and 90 with placebo (room air).

Site: Jinja Hospital Children's Unit (Jinja, Uganda).

Study Duration: 12-18 months (recruitment and acute outcomes); 6 additional months to complete neurocognitive testing of cohort.

Study Participant Duration: 3 days; follow-up neurocognitive testing after 6 months.

Objective: To determine the efficacy of inhaled nitric oxide for the adjunctive treatment of *P. falciparum* malaria.

Our *specific aim* is:

To determine whether supplemental low flow inhaled nitric oxide gas (80 ppm) in addition to Ugandan Standard of Care treatment reduces levels of

angiopoeitin-2 (Ang-2) from baseline in children with moderately severe and severe malaria compared to Standard of Care treatment alone.

The working hypothesis for this aim is that young children hospitalized with malaria will benefit from adjunctive iNO in addition to standard therapy, as determined by more rapid improvement in plasma Ang-2 levels as well as other clinically relevant outcomes. We will test this hypothesis by comparing the daily rate of change of Ang-2 over the hospital admission between the two groups randomized to receive iNO or placebo (primary outcome). We will also compare relevant clinical outcomes including therapeutic efficacy, safety and neurocognitive outcome (secondary outcomes).

1 GENERAL INFORMATION

1.1 Title: Inhaled Nitric Oxide for the Adjunctive Therapy of Severe Malaria: a Randomized Controlled Trial

Draft: 7

Date: 20 Oct, 2010

1.2 Sponsors

1. McLaughlin Rotman Centre for Global Health (MRC)

Toronto General Hospital, University of Toronto

10-401, MaRS building, 101 College St

Toronto, ON, Canada, M5G 1L7

1.3 Name and title of person authorized to sign the protocol and the protocol amendments for sponsor:

Name: Kevin Kain

Title: Director, Sandra A Rotman Labs, MRC

Professor of Medicine, University of Toronto

Director, Centre for Travel and Tropical Disease, University Health Network

1.4 Medical expert for the trial

Name: Kevin Kain

Title: Director, Sandra A Rotman Labs, MRC

Professor of Medicine, University of Toronto

Director, Centre for Travel and Tropical Disease, University Health Network

Address: 200 Elizabeth St., EN 13-214, Toronto, ON, Canada, M5G 2C4

Telephone: 416-340-3535

1.5 Investigators

Principal investigator (Canada):
Prof. Kevin Kain, *MD, FRCPC*

Director, Sandra A Rotman Labs, MRC; Professor of Medicine, University of Toronto; Director, Centre for Travel and Tropical Disease, University Health Network

Principal investigator (Uganda):
Dr. Robert Opika Opoka, MBChB, MMED, MPH

Department of Pediatrics & Child Health, Mulago Hospital

Co-Investigators:

Dr. Abner Tagoola, *MBChB, MMED, MPH*, Jinja Regional Referral Hospital

Dr. Sophie Namasopo, *MBChB, MMED,* Jinja Regional Referral Hospital

Dr. Chandy C. John, *M.D., M.S.*, Department of Pediatrics, University of Minnesota.

Dr. W. Conrad Liles, *MD, PhD,* Dept of Medicine, University of Toronto

Dr. Michael Hawkes, *MD, FRCPC*, Institute of Medical Sciences, Univ of Toronto

Dr. Christopher Miller, *PhD*, University of British Columbia

Trial Site:

Jinja Regional Referral Hospital

Tel +256-434-120622

Fax +256-434-120007

1.6 Physician responsible for all trial-site related medical decisions

Name: Dr. Sophie Namasopo

Title: *MBChB, MMED*

Staff Pediatrician, Jinja Regional Referral Hospital

Address: Nalufenya Road, Jinja, Uganda, Fax +256-434-120007

Telephone: +256-434-120622

1.7 Laboratories and other institutions involved in the trial

1. Makerere University
2. Department of Paediatrics and Child Health, Mulago Hospital, PO Box 7051, Kampala, Uganda, Tel +256 414531875

2 BACKGROUND INFORMATION

Malaria is the leading parasitic cause of morbidity and mortality worldwide, causing an estimated 515 million clinical cases and 2 million deaths annually[1]. Despite the use of highly effective anti-malarial medications, 10-30% patients with severe malaria will die[2], underscoring the need for adjunctive therapies that can be applied in endemic areas. A clinical trial of adjunctive inhaled nitric oxide (iNO) in severe malaria is warranted on the basis of firm proof of concept from animal

models[3] and an uncontrolled human trial using the NO donor L-arginine[4], together with evidence of safety from clinical experience and numerous clinical trials of iNO for other conditions. Here we review the physiologic role of endogenous NO, its role in the pathogenesis and control of infectious diseases and malaria in particular, and the evidence demonstrating decreased bioavailability of NO during severe malaria. Next, we examine the evidence for safety and efficacy of exogenous NO administration, drawing on data from animal models of experimental cerebral malaria and human trials.

2.1 Description of Investigational Product

NO is a gaseous, lipid-soluble free radical that is produced *in vivo* by the enzymatic conversion of L-arginine and molecular oxygen to L-citrulline by members of the NOS family of proteins. Three members of the family have been described to date: neuronal NOS (nNOS or NOS1), inducible NOS (iNOS or NOS2), and endothelial NOS (eNOS or NOS3). Tissue expression of nNOS is primarily in neurons of the brain and peripheral nervous system and eNOS is expressed mainly in epithelial cells, although these isoforms also operate in the immune system [4]. Active NOS is a tetramer of two NOS proteins and two calmodulin molecules. Cofactors for the enzyme include (6R)-tetrahydrobiopterin (BH_4), FAD, FMN and iron protoporphyrin IX (haem).

Both nNOS and eNOS are constitutively expressed and are inactive in resting cells, and are regulated by intracellular calcium flux. Increase in free intracellular calcium levels stabilizes the interaction of calmodulin to nNOS or eNOS and stimulates the

production of NO. This form of regulation leads to transient and short-lasting production of NO, which functions in neuronal signalling and vasodilation. In contrast, iNOS is found in most resting cells, has calcium-independent activity due to tight binding to calmodulin even at low levels of intracellular calcium, and produces high levels of NO for prolonged periods of time. Although all three NOS isoforms have similar NO production rates (~1 μM $min^{-1}mg^{-1}$), iNOS is responsible for high-level production of NO by phagocytes because it is highly expressed after activation[5]. Expression of iNOS in inflammatory and tissue cells is upregulated by exposure to microbial products such as lipopolysaccharide (LPS) and dsDNA or cytokines such as interleukin-1 (IL-1), tumor necrosis factor (TNF) and interferon-γ (IFN-γ)[5, 6]. Enzyme levels are regulated at the level of transcription as well as mRNA stability[5].

Physiologic actions of NO

NO is a highly reactive molecule due to its unpaired electron, mediating a range of effects through three main molecular mechanisms. First, NO reacts readily with transition metals such as iron, copper and zinc, thereby modifying the function of numerous enzymes. Second, NO reacts with thiol groups (e.g., cysteine residues) abundantly present on many proteins to produce S-nitrosothiols, and alters the function of several proteins including $p21^{ras}$, coxsackievirus protease A2, as well as transcription factors, kinases involved in signalling cascades, caspases, ion channels and metabolic proteins[6]. Third, nitric oxide reacts rapidly with superoxide anion (O_2^-) to produce peroxynitrite which is a powerful oxidant, capable of modifying proteins, lipids and nucleic acids. Peroxynitrite plays an important microbicidal role;

however, excessive peroxynitrite formation may lead to cytoxicity through nitration of proteins and inhibition of mitochondrial respiration.

NO has a half-life of several seconds. It can readily diffuse across cell membranes into neighbouring cells, acting as an intercellular messenger. In addition, NO may produce effects distant from its site of production transported by vehicles such as low-molecular weight S-nitrosotiols, S-nitrosylated proteins including haemoglobin and albumin, and nitrosyl-metal complexes which liberate NO spontaneously or after cleavage by ectoenzymes[5]. The activity of NO *in vivo* can be monitored indirectly by measurement of the stable byproducts of NO oxidation, nitrite ($NO2^-$) and nitrate ($NO3^-$), collectively termed NO_x. Dietary factors, exercise and renal insufficiency affect levels of NO_x in body fluids such as plasma and urine, and need to be controlled in comparative studies. Other measures of NO activity are functional (e.g., the NO-mediated reactive hyperaemia peripheral arterial tonometry (RH-PAT) index), or biochemical (e.g., levels of cGMP, a downstream signalling molecule)[7].

One important molecular target of NO is soluble guanylate cyclase (sGC), an enzyme containing a heme moiety with ferrous iron. Formation of a ferrous-nitrosyl-heme complex alters the porphyrin ring structure and leads to activation of sGC with a 400-500 fold increase in the rate of cGMP synthesis[6]. Intracellular signalling by cGMP is mediated to a large extent by cGMP dependent protein kinase (PKG) which promotes smooth muscle relaxation, as well as platelet and neutrophil activation.

Through its action on numerous target molecules, NO is involved in a broad range of physiologic processes. NO was identified originally as the endothelium-derived relaxation factor that mediates vasodilation. Subsequent studies have demonstrated a role for NO in platelet aggregation, endothelial-cell activation, apoptosis, inflammation, chemotaxis, neurotransmission and antimicrobial defense.

Endogenous nitric oxide inhibits the adhesion of platelets to endothelial cells and platelet aggregation[8].

NO in infectious disease

NO plays a complex and versatile role in the pathogenesis and control of infectious diseases. Protective and toxic effects of NO are frequently seen in parallel in the setting of infection because of its variety of molecular targets, widespread production by diverse cell types, and broad capacity for intra- and intercellular signalling[5].

NO attenuates neutrophil respiratory burst and neutrophil-derived oxidative stress[9], and reduces neutrophil rolling and adhesion in microvascular endothelial cells[10]. In adaptive immune responses, NO inhibits T-cell and B-cell proliferation[11]. NO alters cytokine responses, down-regulating pro-inflammatory IL-1, IL-2, TNF and IFN-γ and increasing the production of IL-4, IL-13, and transforming growth factor-β[5].

NO has direct microbicidal activity against numerous viral, bacterial and parasitic agents. The mechanism of action may involve mutation of DNA; inhibition of DNA repair and synthesis; inhibition of protein synthesis; alteration of proteins by S-

nitrosylation, ADP-ribosylation or tyrosine nitration; inactivation of iron, copper or zinc-dependent enzymes; and peroxidation of lipid membranes[5]. Peroxynitrite ($ONOO^-$), a reaction product of NO and superoxide anion (O_2^-), may mediate these effects. In addition to host-derived O_2^- from NADPH oxidase, which has a different temporal pattern of activation within phagocytes than iNOS, pathogen-derived O_2^- may be an important source for $ONOO^-$ formation[5]. Highlighting the central role of $ONOO^-$ in host defense, successful human pathogens, including *M. tuberculosis* and *S. typhimurium*, possess counteracting peroxiredoxins that detoxify $ONOO^-$ to nitrite[12].

NO in malaria
NO plays a role at multiple stages of malaria infection, beginning with the innate defences of the *Anopheles* mosquito vector, where NO protects against the *Plasmodium* parasite[13]. By reversibly binding to salivary proteins (nitrophorins), NO facilitates the mosquito blood meal by enhancing vasodilation and antagonizing hemostasis[14].

Direct anti-parasitic effect
NO has potent microbicidal properties against a number of human pathogens[5]. Exogenous NO did not inhibit the growth of *P. falciparum in vitro*, even at saturating concentrations (2 mM)[15] in one study, but did so in another (Serghides, unpublished data). Byproducts of NO metabolism, including NOx and the nitrosothiol derivatives of cysteine and glutathione, exhibit anti-parasitic activity *in vitro*[15], albeit at concentrations 2 to 3 orders of magnitude higher than those in

human plasma during malaria infection[16]. In experimental murine infection *in vivo*, some studies have found no effect of iNOS deficiency or pharmacologic inhibition of NOS on *P. berghei*[17, 18], *P. chabaudi*[19-22] and *P. yoelii*[23] parasitemia, suggesting a minimal direct anti-parasitic role of endogenous NO, despite important modifying effects on host disease severity[21, 23]. Likewise, in human observational studies, NOx levels are not consistently associated with parasitemia, although they are inversely correlated with disease severity[24, 25].

NO dampens endothelial activation

The vascular endothelium plays a central role in the pathogenesis of cerebral malaria. PEs adhere to the microvascular endothelium resulting in sequestration and vascular obstruction, impaired perfusion and tissue hypoxia[26]. Autopsy studies in fatal cerebral malaria reveal sequestration of PEs in the capillaries and post-capillary venules of multiple organs[27]. Cytoadherence is mediated through constitutive and cytokine-inducible receptors on the endothelial cells, including intercellular cell adhesion molecule-1 (ICAM-1)[28]. NO decreases endothelial cell adhesion molecule expression[29], and has been shown to reduce the adherence of PEs to endothelial cells[30].

Severe malaria is characterized by marked activation of the microvascular endothelium. Circulating levels of adhesion molecules such as ICAM-1 and E-selectin are markedly elevated[30]. Exocytosis of Weibel-Palade bodies (WPB) within endothelial cells releases von Willebrand factor (vWF)[31, 32] and angiopoietin-2 (Ang-2)[33, 34] into the circulation. Ang-2 functions as an autocrine

regulator by sensitizing the endothelium to the effects of TNF, resulting in increased adhesion receptor expression[35]. Vascular endothelial growth factor (VEGF) is another regulator of the vascular inflammatory response, increasing the expression of ICAM-1 and Ang-2 in endothelial cells. Elevated plasma VEGF levels have been associated with increased risk of neurologic sequelae in African children with cerebral malaria[36]. NO inhibits the exocytosis of WPB contents through S-nitrosylation of critical regulatory enzymes[37].

Vasodilation in response to shear stress or chemical agonists is a classical NO-mediated endothelial function that can be monitored non-invasively using reactive-hyperemia peripheral arterial tonometry (RH-PAT)[38]. Severe malaria is characterized by endothelial dysfunction, measured as a subnormal RH-PAT index, which is associated with tissue hypoxia (elevated blood lactate) and hemolysis[4]. In an uncontrolled human trial, endothelial dysfunction was reversible by the administration of L-arginine, a NO donor[4].

NO reduces inflammatory injury in the pulmonary vascular bed

Pulmonary involvement in severe malaria is associated with high mortality, and African children with respiratory distress have a higher risk of death than those with cerebral malaria[39]. Mechanisms of lung injury in malaria share common features with acute lung injury/adult respiratory distress syndrome (ALI/ARDS) in sepsis. Increased alveolar permeability is considered to be the principal functional abnormality underlying ALI/ARDS due to malaria and other causes. Other key events include erythrocyte sequestration and host inflammatory response to parasite

products released into the circulation[40]. Lung histopathologic and ultrastructural studies from individuals with fatal *P. falciparum* infection have found septal and interstital edema, monocytes and parasitized erythrocytes (PE) adherent to the capillary microvasculature, and endothelial cell cytoplasmic swelling[27, 41]. Similarly, in a murine model of experimental malaria-induced lung injury, disruption of the alveolar–capillary membrane barrier and septal inflammation was observed[42]. Thus, the pulmonary vascular endothelium plays a central in ALI during malaria infection, as the site of PE and leukocyte adhesion, and the target of parasite-induced inflammation.

Endogenous NO inhibits inflammatory injury in murine ALI models, as evidenced by increased vascular leak and pathology in $iNOS^{-/-}$ mice[43, 44]. Some studies have documented that iNO decreases pulmonary capillary pressure through selective vasodilatory effects on post-capillary venules[45], and reduces pulmonary edema in patients with ALI[46]. Furthermore, in experimental models of ALI, mice lacking inducible nitric oxide synthase had fewer neutrophils sequestered in the pulmonary vasculature[47], and inhaled NO reduced the accumulation of neutrophils in the pulmonary vasculature and air space[48]. Similar effects of inhaled nitric oxide on leukocyte kinetics are observed outside the lung in rodent models of severe sepsis[49].

Genetic variability in NOS genes and susceptibility to malaria

Natural variation in the genes encoding the NOS enzymes in human populations provides evidence for a protective role of endogenous NO against severe malaria

syndromes. Polymorphisms in the promoter region if the iNOS gene at positions -954 (G-->C) and -1173 (T-->C), located at a putative gene repressor binding site[50], are associated with higher NOS enzymatic activity[50] and higher plasma and urine levels of NOx[24]. These polymorphisms are common in African but not Asian populations[50-53] and are associated with protection from severe malaria in several reports[24, 50, 54]. Another iNOS promoter polymorphism consisting of $CCTTT_{(n)}$ pentanucleotide microsatellite repeats 2.5kb upstream from the iNOS transcription start site has been associated with susceptibility to malaria, although findings from published reports are inconsistent, with long forms of the allele associated with severe disease in some studies[51, 53, 55] but not others[56]. Genetic variation in other NOS isoforms has also been shown to influence the risk of cerebral malaria. A single amino acid substitution at position 298 (Glu-->Asp) in eNOS was associated with increased plasma levels of NOx and protection from cerebral malaria in Indian adults[57]. A single nucleotide polymorphism at position -84 (G-->A) of the nNOS gene is responsible for decreased basal transcriptional level, and is associated with increased risk of cerebral malaria in Indian adults[58]. Taken together, these findings from human population genetic studies indicate that nitric oxide synthase gene polymorphisms affect susceptibility to malaria via alterations in NO production and lend support to a protective role for NO against severe malaria syndromes. Findings from the published reports are summarized in Table 1.

Table 1. Polymorphisms in nitric oxide synthase genes and their association with malaria disease severity.

NOS gene	polymorphism	Population studied	Effect on nitric oxide levels	Susceptibility to malaria	Refs
iNOS promoter	1173 C-->T	Tanzanian children	Increased NOx	Protection against cerebral malaria and severe malarial anaemia	Hobbs, 2002[24]
	954 G-->C	Children in Gabon	7-fold higher baseline NOS activity	Reduced number of malarial attacks<br>Reduced disease severity	Kun, 2001[50]<br>Kun, 1998[54]
	$CCTTT_n$ repeat	Indian and Thai adults, Ghanaian children	NR	Longer repeats associated with severe malaria	Dhangadamajhi, 2009[51]<br>Ohashi, 2002[53]<br>Cramer, 2004[55]
		Gambian children	NR	Shorter repeats associated with severe malaria	Burgner, 1998[56]
		Asymptomatic children and adults, Papua New Guinea	No association between CCTTT repeat number and plasma NOx levels	No association with severe malaria	Hobbs, 2002[24]<br>Boutlis, 2003[52]
eNOS	298 G-->A	Indian adults	Increased NOx	Protection from cerebral malaria	Dhangadamajhi, 2009[57]
nNOS	84 G-->A	Indian adults	Decreased basal transcriptional activity	Increased risk of cerebral malaria	Dhangadamajhi, 2009[58]

NR; not reported

Severe malaria syndromes are characterized by low nitric oxide bioavailability

In a murine model, reduced NO bioavailability contributed to the pathogenesis of ECM[3]. “Footprint” molecules of labile nitric oxide including cGMP and nitrite were markedly decreased and NO supplementation with either a NO donor (dipropylenetriamine NONOate, DPTA/NO) or NO gas provided marked protection against severe disease[3]. Data from human studies supports the hypothesis of reduced bioavailable NO in severe malaria. African children with severe malaria

have impaired production of NO[59], low levels of mononuclear cell iNOS expression[59], and low plasma arginine levels[60], the substrate for NO synthesis.

Factors contributing to reduced bioavailable NO in malaria include scavenging of NO by free haemoglobin and superoxide anion, and reduced levels of NO precursor molecules such as L-arginine and nitrate. Free haemoglobin is released from rupture of parasitized erythrocytes as *Plasmodium* undergoes intraerythrocytic replication and merozoite release, as well as non-specific lysis of uninfected erythrocytes. The ferrous iron within the heme moiety of free haemoglobin reacts with NO, quenching its activity. Experimental evidence in a mouse model confirms elevated levels of plasma oxyheme that increase together with clinical disease score over the course of cerebral malaria, as well as marked quenching of nitric oxide by the plasma of infected mice which was abrogated by cyanine/ferricyanide treatment to inactivate free haemoglobin[3]. In this system, exogenous NO significantly decreased the amount of free haemoglobin, restoring the bioavailable NO pool[3]. Likewise, superoxide anion, released from monocytes and neutrophils during the inflammatory response to *Plasmodium* products such as glycophosphatidylinositol (GPI) and hemozoin/DNA, reacts with and quenches NO [5]. Reduced NO bioavailability has also been demonstrated in other conditions associated with oxidative stress such as ischemia-reperfusion injury[16] and sickle cell anemia[61]. Experimental systems have shown that antioxidants can prevent cerebral malaria in mice infected with *P. berghei*[62]. Reduced levels of the NOS substrate L-arginine have been demonstrated in mice with experimental malaria[3] as well as humans

infected with *P. falciparum*[60]. Release of arginase I from lysed erythrocytes may contribute to hypoargininemia[16]. Treatment of Indonesian adults with severe malaria using intravenous L-arginine reversed malaria-associated endothelial dysfunction and increased levels of exhaled NO[4]. Likewise, nitrite, which is converted to NO by deoxyhemoglobin[63], is reduced in ECM[3] as well as severe malaria in humans[59]. Administration of NO gas restored plasma (but not intra-erythrocytic) nitrite levels in mice[3].

Rationale

Although the use of artemisinin-based antimalarial therapy has improved outcomes in severe malaria, the mortality rates remain high[64]. In a large randomized trial in Southeast Asia comparing artesunate versus quinine, case fatality rates for cerebral malaria were ~30% for those treated with IV artesunate and ~39% for IV quinine[64]. Adjunctive therapies that target the underlying pathophysiology of severe malaria may further reduce morbidity and mortality in severe and cerebral malaria[65].

Previous animal studies have established the beneficial role of inhaled nitric oxide in experimental cerebral malaria[3]. Proof-of-concept trials in Indonesian adults have extended this finding, showing that adjunctive treatment with the NO donor L-arginine accelerates recovery in patients with severe malaria[4]. Based on this proof of concept in animal models and in human disease, and the established safety profile of iNO based on clinical experience and large clinical studies, a trial of iNO as adjuvant therapy for severe malaria is warranted.

2.2 Inhaled nitric oxide in clinical practice and clinical trials

Low-flow iNO at a concentration of 5-80ppm is approved for use by the US FDA for the treatment of neonates with hypoxic respiratory failure. As an adjunctive therapy, it is safe and well tolerated, and is often used in critically ill neonates and infants to treat respiratory distress and meconium aspiration, necrotizing enterocolitis, and persistent pulmonary hypertension of the newborn. In critically ill neonates iNO has been shown to improve oxygenation, improve survival and decrease pulmonary hypertension and requirements for extracorporeal membrane oxygenation (ECMO)[66].

Unlike NO donors such as L-arginine, nitroglycerine and sildenafil, iNO has not been reported to cause systemic vasodilation, electrolyte disturbance or effects on blood glucose. Furthermore, unlike NO donors, inhaled NO does not require functional endothelial cell NO synthase. NOS may be compromised in ill patients and therefore there may be a deficit in their ability to generate NO from these donor molecules. It has been used in a wide variety of clinical settings in adults as well including acute respiratory distress syndrome, pulmonary hypertension, and pregnancy-induced hypertension[67].

Table 2 reviews RCTs of iNO that enrolled and adults and children. Additional trials involving preterm and term neonates are reviewed elsewhere[68]; however, this patient population is not a target population of our proposed RCT. One meta-analysis of 12 trials including 1237 patients with acute lung injury/acute respiratory distress syndrome demonstrated that iNO is generally safe, but was associated with

a statistically significant risk of developing renal dysfunction in these critically ill ICU patients[69].

Table 2: Randomized controlled trials of inhaled nitric oxide.

Reference	*Study design*	*Number of patients*	*Age*	*Target condition*	*Question addressed*	*Findings*	*Dose and delivery route*	*Adverse events*
NINOS. NEJM. 1997.[70]	prospective multicentre controlled RCT	235 (iNO n=114, nitrogen gas n=121)		full-term and nearly full-term infants with hypoxic respiratory failure	evaluate clinical efficacy of iNO in neonates with respiratory failure	iNO reduced the use of extracorporeal membrane oxygenation, but had no apparent effect on mortality	inhaled, 20-80 ppm	Concentration of iNO reduced in 11 patients because of methemoglobinemia (5-10%)
Davidson. Pediatrics. 1998 [71]	prospective multicentre controlled RCT	155 (iNO n=114, nitrogen gas n=41)		Term infants with PPHN	evaluate clinical efficacy of iNO in neonates with respiratory failure	iNO improved oxygenation, no differences in mortality or ECMO	inhaled, 5, 20 and 80 ppm	Elevated methemoglobin (>7%) in 13/37=35% patients receiving 80ppm; and nitrogen dioxide (NO_2>3%) in 7/37=19% patients receiving 80ppm
Dobyns et al. J Pediatr. 1999.[72]	prospective, multicentre, placebo-controlled RCT	108 (placebo gas n=55, iNO n=53)		children, median age 2.5y	determine whether iNO could attenuate progression of lung disease in AHRF, and whether early initation of low-dose iNO results in improved oxygenation		inhaled at 10pp from 3-7 days after entry	metHb none, NO2 high none, no difference in ICU-dependent therapies
Michael JR et al. Am J Resp Crit Care Med. 1998.[73]	prospective, multicentre RCT	40 (no treatment n=20, increasing iNO n=20)	adults and children age 1-79y	beween 12h and 25d after ARDS developed	whether iNO therapy improved oxygenation vs. no treatment within 72hr of randomization, as measured by persistent decreases in FiO2		inhaled at 5, 10, 15, 20 ppm x6h each for 72h	bleeding (1/20 required blood tx, 1/20 ICH after thrombolytic - both iNO groups); no MetHb
Dellinger RP et al. Crit Care Med. 1998.[74]	prospective, phase II, multicentre, placebo-controlled RCT	177 (placebo gas n=57, iNO n=120)	>18yo	non-pregnant, with ARDS not >72h before randomis'n	evaluate safety and physiological effect of iNO, examine response of varied iNO doses in patients with ARDS		inhaled, at doses 1.25, 5, 20, 40, 80 ppm x28d or until extubation	methemoglobin >5% if giving 40+ ppm, hypotension, renal failure,coagulopathy; pneumothorax; all events not sig different between groups

Troncy et al. Am J Resp Crit Care Med. 1998.[75]	prospective, single centre RCT: pilot	30 (no trt n=15, iNO n=15)	adults	AHRF	efficacy of iNO acutely and following prolonged administration, effect on mortality in ARDS		inhaled, increasing doses 2.5, 5, 10, 20, 30, 40 ppm	no MetHb
Lundin S et al. Int Care Med. 1999.[76]	prospective, multicentre, open phase III RCT	268 (iNO n=180, no placebo n=93	adults	ALI with uni- or bilateral lung infiltrates, ventilated 18-96h with high O2	whether early instigation and prolonged therapy of iNO could increase frequency of reversal of ALI	frequency of reversal of ALI no different between iNO and control grps; lower frequency of severe resp failure in iNO; mortality not altered with iNO	NO responders: patients whose PaO2 increased by >20% when receiving 0, 2, 10, 40 ppm iNO x10min within 92h of study entry; responders randomised, iNO 1-40ppm given at lowest effective dose for up to 30d	incr sCr >300 NO 5/80; control 2/74; renal replacement thx NO 23/84 vs control 10/79, RR 2.16. Other events more in NO grp: circ failure, encephalopathy, sesis. No difference in platelet/ coagulopathy
Gerlach H et al. Am J Respir Crit Care Med. 2003.[77]	prospective, single centre RCT	40 (no treatment n=20, iNO 10ppm n=20)	adults 18-64y	ARDS, ventilated >48h with FiO2 >60%, high PEEP, pulm cap wedge P <18mmHg	analyse dose-response characteristics during long-term inhaled NO (day 0, 2, 4)	long-term iNO at 10ppm leads to enhanced sensitivity after several days, does not allow reduction of ventilation parameters. At higher doses (10 and 100 ppm), oxygenation deteriorated and response to iNO disappeared in several pts; no effect of iNO on duration of mechanical ventilation or stay at ICU	10 ppm during inspiration with inbuilt computerised NO application module (prototype Servo 300, Siemens Elema, Sweden)	no MetHb, no NO2 incr; no bleeding, no difference in additional organ dysfxn
Taylor RW et al. JAMA. 2004.[78]	prospective multicentre placebo-controlled RCT	385 (iNO n=192, nitrogen gas n=193)		mod-severe ARDS, onset within 72h of randomization, no sepsis	evaluate clinical efficacy of low-dose iNO in patients with ALI	iNO at 5ppm did not increase #days patients were alive and off assisted breathing; short-term oxygenation improvement that resolved by 48h; similar mortality between groups	inhaled, 5ppm	no diff in all events; no MetHb, no NO2 incr; sCr incr in NO 22/192 vs control 14/193

2.3 Potential risks and benefits

Risks

The subject population for this proposal will consist of 180 Ugandan children with severe *P. falciparum* malaria admitted to hospital. Participants in the experimental arm of the trial will receive iNO by non-rebreather mask or nasal prongs. Nitric oxide has been safely used in clinical practice[79] and in large clinical trials involving critically ill neonates[66, 68], children and adults[67]. Adverse effects attributable to iNO in these studies are rare and include methemoglobinemia, renal insufficiency, and hypotension. Of note, iNO has been used in clinical trials of critically ill adults and children, as well as preterm and term neonates, where the rate of complications is high, although these complications are not attributable to iNO itself, occurring with similar frequency in treatment and control groups. It is used routinely in clinical practice in critically ill patients.

Procedures performed on patients will be part of their routine care. The only difference will be that slightly more blood than is usually will be drawn for study purposes. The risk to the study participants from venipuncture or finger prick blood collection is minimal and consists of possible persistent bleeding, pain or infection. Experienced nurses and medical officers will ensure that all testing is done in a sterile manner.

Benefits

Children with malaria enrolled in the study in both treatment and control arms will receive state-of-the-art diagnosis and treatment for their illness as a direct benefit of study participation. Children with malaria in the iNO arm may benefit directly from the

intervention if iNO is beneficial as adjunctive therapy, as hypothesized. Patents in the control group will have no direct benefit from adjunctive therapy, but will incur minimal risk from this study. The study may be an impetus for increased research in this area, which is a potential long-term benefit of the study to the community but not to the study subjects. If this study documents a treatment benefit of iNO, this would represent a major advance in malaria therapeutics will potential wide-reaching impact for the treatment of malaria globally.

2.4 Justification of route of administration, dosage, dosage regimen, and treatment period

iNO is US FDA-approved for the treatment of hypoxic respiratory failure in term and near-term neonates at a dose of 10-80ppm. We plan to use the upper range of the approved clinical dose to maximize the opportunity to observe a treatment effect, with appropriate monitoring for dose-dependent adverse effects (e.g., methemoglobinemia). iNO, a gaseous molecule, is administered by inhalational route. This route is attractive because of ease of delivery in resource-constrained settings, where IV drug delivery may be challenging. The 72 hour timeframe is based on the period during which iNO will produce the highest likelihood of benefit, since mortality, Ang-2 levels, and endothelial dysregulation are most pronounced in the first few days after admission. Ang-2 levels are elevated at presentation and return to normal levels in survivors over a period of approximately 72 hours[33].

2.5 Statement of compliance

The study will be carried out in accordance with Good Clinical Practice (GCP) as required by the following:

- International Conference on Harmonization (ICH) guideline E6: Good Clinical Practice: Consolidated Guideline

2.6 Description of the population to be studied

Jinja Regional Referral hospital admits at least 175 children with severe malaria (excluding SMA) annually, representing over 30% of total admissions. Malaria transmission is moderate and seasonal in Jinja, which lies in the central area of Uganda, near the capital, Kampala. Resistance to chloroquine and sulfadoxine-pyrimethamine is widespread (34% to 67%)[80].

3 TRIAL OBJECTIVES

The objective of this trial is to determine the efficacy of inhaled nitric oxide (iNO) for the adjunctive treatment of *P. falciparum* malaria.

Our specific aims are:

1. To determine whether supplemental iNO gas (80 ppm) in addition to Ugandan Standard of Care treatment accelerates the rate of decline in angiopoeitin-2 from admission levels in children with severe malaria compared to Standard of Care treatment plus placebo.

2. To determine the clinical efficacy of iNO in severe malaria.

3. To determine the tolerability and safety of iNO in severe malaria.

The working hypothesis is that young children hospitalized with malaria will benefit from adjunctive iNO in addition to standard therapy, as determined by more rapid improvement in serum angiopoietin-2 (Ang-2) levels (an objective and quantitative biomarker of malaria disease severity) as well as other clinically relevant outcomes. We will test this hypothesis by comparing the daily rate of change of Ang-2 over the hospital admission (primary outcome) between the two groups randomized to receive iNO or placebo (room air). We will also compare relevant clinical outcomes including efficacy, safety and tolerability (secondary outcomes).

4 TRIAL DESIGN

4.1 Endpoints

Primary endpoint: Daily rate of change of Ang-2

Secondary endpoints:

1. Clinical efficacy:
    a. 48 hour and 14-day mortality
    b. recovery times:
        i. time to fever resolution
        ii. time to sit unsupported
        iii. time to hospital discharge
    c. time to parasite clearance
    d. incidence of recrudescence at day 14

2. Other biomarkers and genetic determinants of malaria disease severity
    a. whole blood lactate level
    b. biomarkers and genetic determinants of endothelial activation, inflammation and coagulopathy, to be determined
3. Neurocognitive outcome at 6 months after discharge

Primary outcome

The daily change in Ang-2 over the hospital admission will be the primary efficacy outcome. Elevated Ang-2 levels are associated with poor clinical outcome in severe malaria[33, 34] and Ang-2 has been used to follow disease progression and recovery in previous studies of malaria[33]. Thus, Ang-2 is an objective, quantitative marker of disease severity, validated for longitudinal follow-up of patients with malaria. Ang-2 levels will be measured longitudinally at admission (day 0), day 1, day 2 and day 3 of hospital admission. In patients treated for severe malaria who survive the infection, Ang-2 levels have been shown to decrease linearly during recovery at a mean rate of 2700pg/mL per 24h[33].

Secondary outcomes

Therapeutic efficacy

We will examine the efficacy of adjunctive iNO treatment using established clinical and parasitological outcomes: in-hospital mortality, recovery times (time to fever resolution, time to sit unsupported, and time to hospital discharge), time to parasite clearance, incidence of parasite recrudescence at day 14 after hospital discharge. These are standard outcomes in other therapeutic trials for malaria[64, 81, 82]. Routine intensive

monitoring of vital signs, clinical assessments on at least a daily basis by physicians experienced in the management of severe malaria, as well as measurement of key laboratory parameters and parasite density will be performed to collect this data.

Biomarkers and genetic determinants of disease severity

Lactate is produced by the anaerobic metabolism of glucose in the absence of adequate tissue oxygenation. As such, elevated lactate levels represent a final common pathway of tissue hypoxia and decompensated shock, the forerunner of cardiovascular collapse and death. Lactate levels at presentation predict pediatric mortality in a wide variety of clinical conditions including malaria[83, 84], sepsis[85], pediatric trauma[86] and cardiac disease[87]. We will measure lactate as an independent biomarker of disease severity during the clinical trial.

Biomarkers and genetic determinants of severe malaria pathogenesis may provide additional insight into the pathways and processes altered in cerebral malaria and affected by iNO delivery. We plan to examine biomarkers of endothelial activation, inflammation including cytokines, and coagulopathy which are central to the pathophysiology of severe malaria. In addition, genetic pathways involved in severe malaria and response to iNO will be investigated.

Neurocognitive outcome

In order to establish whether iNO improves long-term neurocognitive outcomes in children with severe malaria, we will also establish the areas, frequency and severity of cognitive and neurologic function affected by cerebral malaria in children of different

ages up to age 5. The overall cognitive deficit at 6 months after discharge will be assessed by doing neuropsychological tests as described in SOPs.

4.2 Trial design

Prospective, parallel arm, randomized, placebo-controlled, double-blind clinical trial.

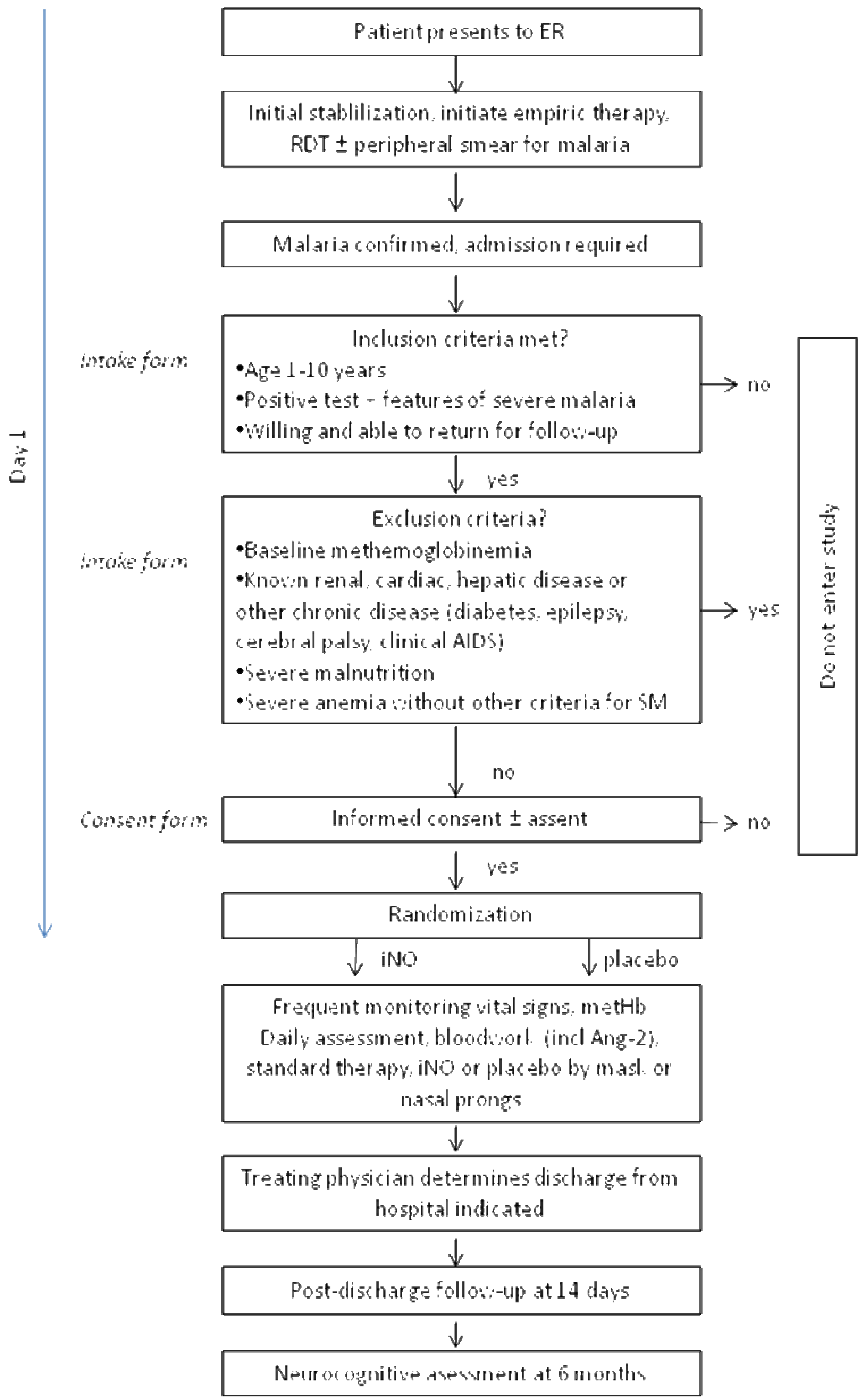

Figure 1. Flow diagram of patient evaluation, enrolment, randomization, and measurement throughout the trial.

4.3 Description of measures taken to minimize bias

(a) Randomization

Sequence generation

A computer generated randomization list will be created. Simple randomization will be used. Important prognostic variables including patient age, disease severity, and HIV seropositivity will thus be expected to be distributed randomly between treatment arms.

Allocation concealment

After informed consent/assent is obtained, a study site investigator will draw a sequentially numbered sealed opaque envelope. The investigator will sign and date across the seal then open it to reveal randomization arm (iNO or placebo). Hence, local investigators will be unaware of the allocated treatment until after the patient has been randomized. We will retain all envelopes and records for subsequent inspection by study monitors.

(b) Blinding

In previous clinical trials using iNO[69], one of the design and implementation challenges was establishing the blinding procedures while titrating and monitoring concentrations of iNO, as well as anticipated dose-related increases of methemoglobin and NO_2 concentrations. A trial manager not involved in patient care will be responsible for the monitoring and recording of iNO, NO_2, and methemoglobin levels. Thus, the establishment of two teams, one blinded team making all clinical assessments and therapeutic decisions, and another unblinded team monitoring the delivery of the treatment gas and assessing the development of potential toxicities, will allow the drug

to be delivered safely while minimizing the possibility that direct knowledge of treatment allocation would influence the care delivered to the patient.

iNO cylinders will be attached to ventilation system in all patients. The unblinded study site investigator will administer and monitor the delivery of NO or placebo (room air), according to the treatment arm to which the patient has been randomized.

4.4 Investigational treatment

Product: Gaseous nitric oxide

Dose: 80 ppm

Dosing schedule: Continuous

Route: Inhalation (non-rebreather HiOx® face mask)

Treatment period: Maximum 72 hours (may be discontinued earlier if patient recovers and no longer tolerates face mask)

The iNO will be supplied in cylinders that will be appropriately labelled.

4.5 Expected duration of subject participation

Duration of study gas (iNO or room air placebo): maximum 72 hours (may be discontinued earlier if patient recovers and no longer tolerates face mask)

Expected total duration of hospitalization for severe malaria: 5 to 7 days

Follow-up (1st visit): 14 days after admission

Follow-up (2nd visit): 6 months after admission

4.6 Description of stopping rules/discontinuation criteria

Individual patients

We distinguish between adverse events that will result in temporary discontinuation of the study gas with the possibility of re-challenge after return to normal levels, and adverse events that will lead to patient withdrawal from the study.

Temporary discontinuation of treatment

Treatment with study gas (iNO or placebo) will be temporarily discontinued if the following adverse events occur:

- Methemoglobinemia
- Elevated inspired NO_2 concentration
- Persistent hypoxemia
- Evolving respiratory distress
- Unexplained tachycardia
- Unexplained hypotension
- Any study drug related adverse event that, in the opinion of the investigator, makes it unsafe for the subject to continue

Details and operational definitions are provided in the SOP: *Discontinuation of study gas*.

Subjects may be rechallenged with study gas (iNO or room air) after metHb, SpO_2, HR, or SBP return to normal levels after discontinuation of study gas (iNO or room air).

Permanent Discontinuation of treatment

If a subject experiences any of the following events during the 3 day iNO delivery period the study gas should be permanently discontinued:

- Refractory methemoglobinemia
- Hemoptysis

- Acute kidney injury
- Any study drug related adverse event that, in the opinion of the investigator, makes it unsafe for the subject to continue
- Any study drug related adverse event that repeats upon re-challenge at the same or lower dose level
- At the discretion of the subject or guardian
- At the discretion of the Investigator, if deemed appropriate, for any reason

Patients meeting these criteria will have study gas removed, and will be continued to be monitored until resolution of the abnormality. When study gas is permanently discontinued, patient data will continue to be collected for primary and secondary trial endpoints, including daily bloodwork. These results will be included in an intention-to-treat analysis.

Entire trial

Halting rules temporarily suspend study enrolment until a safety review is convened. The objective of this review will be to decide whether the study should continue per protocol, proceed with caution, be further investigated, be discontinued or be modified and then proceed.

Halting rules for this study will be:

1. Mortality in any sequential group of 10 patients above statistical upper control limit (6/10 patients = 60%).
2. Cumulative mortality in any of the arms of the trial in excess of the upper statistical threshold.

Justification for these statistical thresholds is given in Appendix 1.

Safety review, interpretation of results and decisions about discontinuation of the trial will be made by a Data and Safety Monitoring Board (DSMB), using the suggested guidelines given here. We do not propose that the DSMB be strictly bound by pre-specified criteria, because of the complexity of the trade-offs between safety, efficacy, and costs, and the possibility that new information will change considerations. Rather, consideration of stopping guidelines requires a reasoned judgment based on all information that is available at the time of data review.

4.7 Accountability procedures, including placebo and comparators

iNO will be compared head-to-head with room air placebo in a randomized, parallel group design. All patients will receive standard-of-care antimalarial agents and supportive therapy for their severe malaria. Treatment and placebo will be indistinguishable for participants, caregivers and health professionals. All patients will wear a mask and receive room air, with supplemental oxygen as needed, delivered via compressor. The treatment group will also receive iNO mixed with the air flow. The dilution will be such that there will not be a perceptible difference between treatment and placebo flow rates.

4.8 Maintenance of the trial treatment randomization codes and procedures for breaking codes

The need for continuous safety monitoring (NO and NO_2 levels in inhaled gas, methemoglobin levels) requires the presence of an unblinded team. This team will consist of the trial manager and locum nurses to assist with night coverage. The randomization code will be kept in a locked cabinet accessible by the trial manager only. The code will be consulted at the point of randomization, at which point the iNO or room

air placebo will be started by the trial manager. The code will be used for the purpose of tabulating data for the DSMB review on a quarterly basis, and at the midpoint of patient enrolment. Data will be disaggregated by treatment arm, although the treatment received (iNO or placebo) will not be known to the DSMB ("Group A" and "Group B").

Unblinding of individual patients may be required under exceptional circumstances if there are safety concerns. The Jinja pediatrician may request this information from the trial manager in the rare event that knowledge of study gas may influence patient care. The entire trial population will be unblinded after completion of enrolment. Statistical analysis will be conducted in a blinded fashion, with patients assigned to "Group A" of "Group B" by the trial manager.

We will assess whether blinding has been successful by asking key trial persons (caregivers, health professionals, outcome assessors) to guess patients' treatments and compare the answers with the actual treatments[88]. Statistical differences from chance alone will be evidence that trial blinding was compromised.

4.9 Data to be included on the case report forms (CRF)

Study participants

After initial stabilization, participants recruited to the trial will have a screening form completed, according to inclusion and exclusion criteria. Patient age, co-morbidities, nutrition status (weight and height), malaria rapid diagnostic test result, anemia, methemoglobin level, and willingness to participate in the trial will be documented on the screening form and will be the basis of deciding on whether a child is eligible for the study. Eligible, consenting participants will then undergo a full history and physical

examination, the results of which will be recorded on the CRF. Study entry blood work will include the following, and a checklist on the CRF will be completed to ensure that the appropriate specimens have been collected for laboratory testing:

- CBC and differential
- Electrolytes
- Creatinine
- Lactate
- Venous blood gases
- Blood culture
- CSF gram stain and culture (as appropriate)
- CSF cell count and differential (as appropriate)
- HIV point-of-care test
- Serum and plasma sample for Ang-2

Primary endpoint: Daily rate of change of Ang-2

Four venous blood samples, appropriately collected, aliquoted, and stored are required for this endpoint. The CRF will document the successful collection of venous samples on a daily basis over the course of the study, and any problems that may compromise the sample quality. In the case of missing/incomplete data, the CRF will document the reason for missing data.

Secondary endpoints:

48 hour and 14-day mortality

The CRF will document whether the patient was alive or deceased or lost to follow-up at 48 hours after admission, and at the 14-day follow-up visit.

Time to fever resolution

Trial nurses will document vital signs on the CRF (every 4 hours for the first day, every 8 hours thereafter). The time to achieve a temperature <38°C for ≥24 hours will be derived from these data.

Time to sit unsupported

Trial nurses will document at the end of each shift whether the patient is able to sit unsupported and the time to achieve this endpoint will be derived from these data.

Time to hospital discharge

CRF completed by the medical officers will include the admission and discharge dates.

Time to parasite clearance

Parasite density will be followed longitudinally over the course of the hospital admission. The CRF will document that a peripheral sample has been obtained for parasitemia.

Incidence of recrudescence at day 14

The 14-day follow-up visit will document that a peripheral blood sample has been obtained for determination of parasite density.

Whole blood lactate level

A handheld lactate meter will be used by medical officers to document whole blood lactate levels at each blood draw. The CRF will record the lactate levels, the time of collection, and any difficulties that may compromise the validity of the data.

Neurocognitive outcome at 6 months after discharge

CRFs for the neurocognitive outcome will document findings of testing.

Safety

Adverse events

Adverse events will be recorded on the patient CRF as well as a log tabulating the cumulative adverse events. The adverse events will be described by the nature of the adverse event, date of onset, date of resolution, possible or probable causal association with study gas, severity, and expectedness.

Adverse drug reactions

A checklist of adverse drug reactions (based on product monograph) will be completed on a daily basis by medical officers. This will require a daily review of patient notes during the admission, including vital signs. Age specific norms will be used to classify vital signs as normal or abnormal. The following adverse drug reactions that occurred in at least 5% of patients receiving INOmax in the CINRGI study with event rates >5% and greater than placebo event rates[89]:

- Hypotension
- Withdrawal
- Atelectasis
- Hematuria
- Hyperglycemia
- Sepsis
- Infection
- Stridor
- Cellulitis
- Other (specify)

In the event that the study gas is discontinued or withdrawn (section 5.3 Subject withdrawal criteria), the date, time and reason will be documented on the CRF as well as a dedicated form (checklist) for study gas discontinuation/withdrawal. This form will also document the resolution of the abnormality (vital signs, methemoglobin, oxygen

level) and any sequellae or complications. If the patient is re-challenged with study gas, the timing will be recorded. Criteria for temporary discontinuation are as follows:

- Methemoglobinemia
- Elevated inspired NO_2 concentration
- Persistent hypoxemia
- Evolving respiratory distress
- Unexplained tachycardia
- Unexplained hypotension
- Any study drug related adverse event that, in the opinion of the investigator, makes it unsafe for the subject to continue

Criteria for permanent discontinuation of study gas are as follows:

- Refractory methemoglobinemia
- Hemoptysis
- Acute kidney injury
- Other adverse event that, in the opinion of the investigator, makes it unsafe for the subject to continue.
- Adverse event that repeats upon re-challenge
- Subject or guardian's discretion
- Investigator's discretion

Monitoring for these adverse drug reactions will require laboratory investigations including glucose and creatinine, measured on at least a daily basis. The CRF will include laboratory data (point-of-care test results).

Severe adverse events

Severe adverse events, including mortality, will be recorded on the CRF, and a dedicated SAE report form. The expectedness and possible or probable causal link to the study gas will be judged by medical officers. The SAE forms will be reported to the trial PIs within 24 hours. Of note, significant mortality (estimated 20%) is expected in this trial of critically ill children with severe malaria. Unexpected SAEs will be reported to

MUREC and the DSMB within 15 days (if not life-threatening) and within 7 days in the case of a life-threatening unexpected SAE. As an additional measure, mortality events will be graphically recorded in real time with every consecutive group of 10 enrolled patients. Statistical thresholds have been defined to trigger a safety review if mortality is above expected levels. Quarterly reports of the cumulative trial mortality by study group will be provided to the DSMB.

Quality

Intervention

Administration of study gas will be directly supervised by dedicated trial nurses, who will record the time the patient receives the gas during each shift (8h period) on the CRF. Because patients may not tolerate a mask after partial recovery, the time on the study gas may be less than 72 hours.

Protocol deviations

Protocol deviations will be noted on the CRF as well as a central log.

Blinding

We will assess whether blinding has been successful by asking key trial persons (caregivers, health professionals, outcome assessors) to guess patients' treatments and compare the answers with the actual treatments[88]. These will be recorded on the CRF.

5 SELECTION AND WITHDRAWAL OF SUBJECTS

5.1 Inclusion criteria

1. Age 1-10 years

2. Positive malaria rapid diagnostic test in the presence of selected features of severe malaria[1].
3. Willing and ability to complete follow up schedules for the study – 14 day and 6 months after hospital discharge.

5.2 Exclusion criteria

1. Baseline methemoglobinemia (>2%)
2. Known renal, cardiac, or hepatic disease or other chronic illnesses like diabetes, epilepsy, cerebral palsy, clinical AIDS, suspected or known hemoglobinopathy (e.g., sickle cell anemia, G6PD deficiency).
3. Suspected bacterial meningitis.
4. Patient already received parenteral quinine in the ED prior to eligibility assessment.
5. Patient unlikely to tolerate delivery mask for more than 24 hours.
6. Severe malnutrition
7. Severe malarial anemia (Hb <50g/L) without other signs of severe malaria.

[1] repeated seizures (two or more generalized seizures in 24 h); prostration (in children 1 year and older, the child is unable to sit unsupported or stand although was able to before the illness; impaired consciousness (Blantyre Coma Score <5 in children 1 to 4 years, GCS <14 for children ≥ 5 years); respiratory distress: sustained nasal flaring, deep breathing or intercostal/subcostal retractions.

5.3 Subject withdrawal criteria (terminating trial treatment)

(a) When and how to withdraw subjects from the trial

We distinguish between adverse events that will result in temporary discontinuation of the study gas with the possibility of re-challenge after return to normal levels, and adverse events that will lead to patient withdrawal from the study.

Temporary discontinuation of treatment

Treatment with study gas (iNO or placebo) will be temporarily discontinued if the following adverse events occur:

- Methemoglobinemia
- Elevated inspired NO_2 concentration
- Persistent hypoxemia
- Evolving respiratory distress
- Unexplained tachycardia
- Unexplained hypotension
- Any study drug related adverse event that, in the opinion of the investigator, makes it unsafe for the subject to continue

Details and operational definitions are provided in SOP: discontinuation of study gas.

Subjects may be rechallenged with study gas (iNO or room air) after metHb, SpO_2, HR, or SBP return to normal levels after discontinuation of study gas (iNO or room air).

Permanent Discontinuation of treatment

If a subject experiences any of the following events during the 3 day iNO delivery period the study gas should be permanently discontinued:

- Refractory methemoglobinemia
- Hemoptysis
- Acute kidney injury

- Any study drug related adverse event that, in the opinion of the investigator, makes it unsafe for the subject to continue
- Any study drug related adverse event that repeats upon re-challenge at the same or lower dose level
- At the discretion of the subject or guardian
- At the discretion of the Investigator, if deemed appropriate, for any reason

Patients meeting these criteria will have study gas removed, and will be continued to be monitored until resolution of the abnormality. When study gas is permanently discontinued, patient data will continue to be collected for primary and secondary trial endpoints, including daily bloodwork. These results will be included in an *intention-to-treat* analysis.

(b) **The type and timing of data to be collected for withdrawn subjects:**
If for any reason a subject does not complete the study, the reason will be entered on the CRF. All subjects are free to withdraw from participation at any time, for any reason, specified or unspecified, and without prejudice. The reason for the early termination will be recorded. The time to resolution of the abnormal result, and any clinical sequellae must also be recorded.

Patients for whom study gas is permanently discontinued will continue to have data collected for primary and secondary trial endpoints, including daily bloodwork. Their results will be included in an intention-to-treat analysis.

(c) **Whether and how subjects are to be replaced:**
At the midway point of recruitment (90 patients enrolled), an assessment will be made of the number of patients with complete and accurate evaluable data. The current

sample size is estimated for a dropout rate of 10%. If treatment discontinuation and/or withdrawal exceeds 10%, a sample size recalculation will be considered to adjust for greater than expected loss of evaluable patients without compromising study power.

(d) **The follow-up for subjects withdrawn from trial treatment**

If a subject is withdrawn because of an adverse event, the subject will be followed and treated by the medical officer until the abnormal parameter or symptom has resolved or stabilized.

The adverse events must be followed to resolution and the follow-up evaluations noted in Adverse Event CRF and recorded when the subject has stabilized.

6 TREATMENT OF SUBJECTS

6.1 Description of intervention

Product: Gaseous nitric oxide

Dose: 80 ppm

Dosing schedule: Continuous

Route: Inhalation (non-rebreather HiOx® face mask)

Treatment period: Maximum 72 hours (may be discontinued earlier if patient recovers and no longer tolerates face mask)

Gaseous NO (delivered with air and oxygen as carrier) at a concentration of 80 parts per million (ppm) will be administered continuously by inhalation (non-rebreather HiOx®

face mask) for a maximum of 72 hours. Subjects in the control arm will receive room air as a placebo for a maximum of 3 days. The iNO dose of 80 parts per million is US-FDA approved for use in neonates (see *INOmax product monograph*).

To monitor the inspired nitric oxide, nitrogen dioxide (NO_2) and oxygen concentrations, a US-FDA approved gas monitor will be used. Should the NO or NO_2 concentrations fall outside the targeted range, an operator set alarm will sound. The trial manager or designee (unblinded) will be available on a 24 hour basis to respond to monitor alarms.

Nitric oxide will be delivered as long as possible with the above device, up to a maximum of 72 hours. However, as the subject improves in the course of the disease, recovers consciousness, begins ambulating, and/or no longer tolerates the mask delivery system, the study gas may be discontinued earlier than 72 hours. At this point after significant disease resolution, it is expected that the continued benefit of iNO will be negligible. The trial manager or designee (unblinded) will be responsible for initiating the study gas (iNO or room air placebo), monitoring the inhaled NO, NO_2 and O_2 levels as well as patient methemoglobin levels, responding to any monitor alarms, initiating corrective actions, and informing the treating physicians, if necessary. We will monitor peripheral oxyhemoglobin saturation, and use the minimum inspired O_2 concentration (above 21%) required to maintain a PaO2≥92%.

As a primary safety measure, every patient will be monitored with a US-FDA approved pulse CO-oximeter for methemoglobin and arterial oxygen saturation. This will assure, metabolically, that too much nitric oxide is not being inhaled and that the inspired oxygen concentration is correct.

Each subject will receive their own mask to avoid cross contamination. During study gas treatment, a nurse will be present at all times. A physician (medical officer) will follow patients daily, and will be on call on a 24 hour basis. Nurses and physicians will be trained in the clinical recognition of methemoglobinemia and its management before commencement of the trial. Thresholds for temporary discontinuation of study gas or withdrawal from the study based on methemoglobin measurements are given in section 5.3 *Subject withdrawal criteria*.

Both treatment arms (iNO and room air placebo) will receive standard antimalarial therapy and supportive measures according to Ugandan national guidelines.

6.2 Co-administered treatments during the trial

All patients in the trial will receive Uganda standard of care treatment for severe malaria. This will include anti-malarial therapy (generally intravenous quinine), supportive therapy with intravenous fluids, blood transfusion, glucose, antipyretics, and anticonvulsants as needed, and treatment for suspected bacterial co-infection with broad-spectrum antibiotics at the treating physicians discretion. There is no restriction on medications/treatments for patients in the trial.

6.3 Procedures for monitoring subject compliance

Patients will be directly observed in the hospital by dedicated trial nurses. The time that they remain on the study gas will be recorded each shift. In the event that the mask is

not tolerated by children in the study, this will be recorded in the nursing record (case report form).

7 ASSESSMENT OF EFFICACY

7.1 Specification of efficacy parameters

Primary endpoint: Daily rate of change of ANG-2

Secondary endpoints:

1. Clinical efficacy:
    a. 48 hour and 14-day mortality
    b. recovery times:
        i. time to fever resolution
        ii. time to sit unsupported
        iii. time to hospital discharge
    c. time to parasite clearance
    d. incidence of recrudescence at day 14
2. Other biomarkers and genetic determinants of malaria disease severity
    a. whole blood lactate level
    b. biomarkers and genetic determinants of endothelial activation, inflammation and coagulopathy
3. Neurocognitive outcome at 6 months after discharge

Primary outcome

The daily change in Ang-2 over the hospital admission will be the primary efficacy outcome. Elevated Ang-2 levels are associated with poor clinical outcome in severe malaria[33, 34] and Ang-2 has been used to follow disease progression and recovery in previous studies of malaria[33]. Thus, Ang-2 is an objective, quantitative marker of disease severity, validated for longitudinal follow-up of patients with malaria. Ang-2 levels will be measured longitudinally at admission (day 0), day 1, day 2 and day 3 of hospital admission. In patients treated for severe malaria who survive the infection, Ang-2 levels have been shown to decrease linearly during recovery at a mean rate of 2700pg/mL per 24h[33].

Secondary outcomes

Therapeutic efficacy

We will examine the efficacy of adjunctive iNO treatment using established clinical and parasitological outcomes: in-hospital mortality, recovery times (time to fever resolution, time to sit unsupported, and time to hospital discharge), time to parasite clearance, incidence of parasite recrudescence at day 14 after hospital discharge. These are standard outcomes in other therapeutic trials for malaria[64, 81, 82]. Routine intensive monitoring of vital signs, clinical assessments on at least a daily basis by physicians experienced in the management of severe malaria, as well as measurement of key laboratory parameters and parasite density will be performed to collect this data.

Biomarkers and genetic determinants of disease severity

Lactate is produced by the anaerobic metabolism of glucose in the absence of adequate tissue oxygenation. As such, elevated lactate levels represent a final common pathway

of tissue hypoxia and decompensated shock, the forerunner of cardiovascular collapse and death. Lactate levels at presentation predict pediatric mortality in a wide variety of clinical conditions including malaria[83, 84], sepsis[85], pediatric trauma[86] and cardiac disease[87]. We will measure lactate as an independent biomarker of disease severity during the clinical trial.

Biomarkers and genetic determinants of severe malaria may provide additional insight into the pathways and processes altered in cerebral malaria and affected by iNO delivery. We plan to examine biomarkers of endothelial activation, inflammation including cytokines, and coagulopathy which are central to the pathophysiology of severe malaria. In addition, genetic pathways involved in severe malaria and response to iNO will be investigated.

Neurocognitive outcome

In order to establish whether iNO improves long-term neurocognitive outcomes in children with severe malaria, we will also establish the areas, frequency and severity of cognitive and neurologic function affected by cerebral malaria in children of different ages up to age 5. The overall cognitive deficit at 6 months after discharge will be assessed by doing neuropsychological tests as described in SOPs.

7.2 Methods and timing for assessing, recording, and analyzing efficacy parameters

Clinical outcomes will be assessed daily by trial nurses and medical officers. They will be recorded on standardized case report forms. The data will be entered into a computerized database. Statistical analysis for the primary endpoint is described in

section 9.1 *Statistical methods*. In brief, a mixed-effects linear model will be used to determine differences in the change of Ang-2 over the first 72 hours of hospital admission.

8 ASSESSMENT OF SAFETY

Adverse events may occur commonly in a trial involving children with severe malaria, although the majority of events are likely due to the clinical course of malaria and not to study medications. Mortality in children at nearby Mulago Hospital with severe malaria was as high as 16% among children with impaired consciousness and 21% among children with deep acidotic breathing as presenting clinical signs[80].

8.1 Specification of safety parameters

The following standard definitions are based on the International Committee on Harmonization Guideline on Clinical Safety Data Management.

Adverse Event (or Adverse Experience)

Any untoward medical occurrence in a patient or clinical investigation subject administered a pharmaceutical product and which does not necessarily have to have a causal relationship with this treatment.

An adverse event (AE) can therefore be any unfavourable and unintended sign (including an abnormal laboratory finding), symptom, or disease temporally associated with the use of a medicinal product, whether or not considered related to the medicinal product.

In this trial, a running log of adverse events will be kept and reviewed periodically to allow the study team to assess if any patterns in adverse events emerge in real time.

Adverse Drug Reaction (ADR)

Inhaled nitric oxide has been used in clinical practice and in several clinical trials, and is a marketed medicinal product. Regarding *marketed medicinal products*, a well-accepted definition of an adverse drug reaction in the post-marketing setting is:

A response to a drug which is noxious and unintended and which occurs at doses normally used in man for prophylaxis, diagnosis, or therapy of disease or for modification of physiological function.

The iNO product monograph defines the following adverse reactions that occurred in at least 5% of patients receiving INOmax in the CINRGI study with event rates >5% and greater than placebo event rates[89].

Table 3. Adverse events associated with the use of iNO.

**Adverse Event**	**Placebo (n=89)**	**Inhaled NO (n=97)**
Hypotension	9 *(10%)*	13 *(13%)*
Withdrawal	9 *(10%)*	12 *(12%)*
Atelectasis	8 *(9%)*	9 *(9%)*
Hematuria	5 *(6%)*	8 *(8%)*
Hyperglycemia	6 *(7%)*	8 *(8%)*
Sepsis	2 *(2%)*	7 *(7%)*
Infection	3 *(3%)*	6 *(6%)*
Stridor	3 *(3%)*	5 *(5%)*
Cellulitis	0 *(0%)*	5 *(5%)*

In addition, the following adverse drug reactions have been reported during post-approval use of iNO: dose errors associated with the delivery system; headaches associated with environmental exposure of iNO in hospital staff; hypotension associated with acute withdrawal of the drug; hypoxemia associated with acute withdrawal of the drug; pulmonary edema in patients with CREST syndrome. These reactions are reported voluntarily from a population of uncertain size; therefore, it is not always possible to estimate their frequency reliably or to establish a causal relationship to drug exposure.

For the purposes of safety monitoring and reporting, adverse drug reactions will include temporary as well as permanent discontinuation of the study gas. Criteria have been established for temporary and permanent study gas discontinuation, and are listed in sections 4.6 Description of stopping rules/discontinuation criteria, and 5.3 Subject withdrawal criteria (terminating trial treatment).

Unexpected Adverse Drug Reaction
An adverse reaction, the nature or severity of which is not consistent with the applicable product information (Product Monograph – see Table 3 for expected adverse drug reactions).

Adverse reactions outside the range or severity of those listed in Table 3 and judged to be related to the study medication will be considered unexpected adverse drug reactions. Medical officers, the Jinja pediatrician co-investigator may judge the likelihood that an adverse event is related to study medication and is outside the

expected spectrum of previously documented adverse drug reactions for the purposes of reporting.

Serious Adverse Event or Serious Adverse Drug Reaction

A serious adverse event (experience) or reaction is any untoward medical occurrence that at any dose:

- *results in death,*
- *is life-threatening,*
- *requires prolongation of existing hospitalisation, or*
- *results in persistent or significant disability/incapacity*

In this trial of critically ill children hospitalized with severe malaria, mortality is expected to occur in approximately 20% of participants who receive standard medical care[90]. Monitoring serious adverse events during the trial is therefore complicated by the fact that death is part of the natural progression of disease, even under conditions of optimal clinical care (Uganda standard of care). We propose to monitor mortality continuously to detect variations above the baseline expected mortality rate, to allow for early implementation of corrective actions, if required.

8.2 The methods and timing for assessing, recording, and analysing safety parameters

Adverse events, ADRs, and SAEs will be monitored continuously during the trial by medical officers and/or dedicated nursing staff. They will be recorded on case report forms. In addition, a running log of adverse events will be kept and reviewed periodically to allow the study team to assess if any patterns of adverse events emerge in real time. Discontinuation of study gas treatment and/or withdrawal of study

treatment in any subject will require the completion of a form detailing the reason for study gas discontinuation/withdrawal. All study deaths will require completion of an SAE form, which will be made available to the DSMB, ethics boards and regulatory authority according to their requirements, as described in section *8.3 Procedures for eliciting reports of and for recording and reporting adverse event and intercurrent illnesses*.

Halting rules

Halting rules temporarily suspend study enrollment until a safety review is convened. The objective of this review will be to decide whether the study should continue per protocol, proceed with caution, be further investigated, be discontinued or be modified and then proceed.

Halting rules for this study will be:

1. Mortality in any sequential group of 10 patients above statistical upper control limit (6/10 patients = 60%).
2. Cumulative mortality in any of the arms of the trial in excess of the upper statistical threshold.

Justification for these statistical thresholds is given in Appendix 1.

Safety review, interpretation of results and decisions about discontinuation of the trial will be made by a Data and Safety Monitoring Board (DSMB), using the suggested guidelines given here. We do not propose that the DSMB be strictly bound by pre-specified criteria, because of the complexity of the trade-offs between safety, efficacy,

and costs, and the possibility that new information will change considerations. Rather, consideration of stopping guidelines requires a reasoned judgment based on all information that is available at the time of data review.

8.3 Procedures for eliciting reports of and for recording and reporting adverse event and intercurrent illnesses

SAE forms will be made available to the DSMB, ethics boards and regulatory authority according to their requirements, which differ between each body. Accordingly, all SAEs will be reported to MUREC and the Uganda NDA as soon as possible, but within 15 days of the event. Unexpected SAEs will additionally be reported to the DSMB chairperson, MUREC, NDA, Jinja Hospital REB (as soon as possible, but within 15 days of the event). Fatal and life-threatening unexpected SAEs will be reported to the DSMB chairperson, MUREC, NDA, Jinja Hospital REB as soon as possible, but within at most 7 calendar days. Finally, SAEs that are unexpected, related or possibly related to the study intervention, and suggest an increase in risk to study participants or others will also be reported to the UHN REB (Toronto).

All SAEs that occur during hospitalization will be reported as described above. Regarding SAEs that occur after hospital discharge, reports will be issued as described above for events occurring up to day 14 of the study (i.e., after hospital discharge, out to 14 days post-enrolment).

8.4 The type and duration of the follow-up of subjects after adverse events

If a subject is withdrawn because of an adverse event, the subject will be followed and treated by the medical officer until the abnormal parameter or symptom has resolved or stabilized.

The adverse events must be followed to resolution and the follow-up evaluations noted in Adverse Event CRF and recorded when the subject has stabilized.

9 STATISTICS

9.1 Description of statistical methods to be employed

Primary outcome

The primary focus is whether or not the rate of reduction in Ang-2 differs between the treatment groups. In statistical terms, this is a *time-by-treatment* interaction. Given that we will have repeated measurements, possibly incomplete, over time, a linear mixed-effect model will be used to estimate and test the magnitude of the *time-by-treatment* interaction. A linear time trend will not be assumed by treating "day" as a categorical variable in the model. Log transformation of the data may be considered, as appripriate.

The primary analysis will be by *intention-to-treat*. That is, patients will be analysed in the group to which they were randomized, regardless of the deviations from study protocol. A secondary *per-protocol* analysis may be considered if important deviations from protocol compromise the validity of the ITT analysis. Subgroup analyses related to important prognostic factors will be performed: age < 5 years or ≥5 years;

uncomplicated or severe malaria; HIV seropositivity; and bacterial co-infection (see below).

Secondary outcomes

Mortality. Analysis will follow standard methods in other clinical trials for malaria[64, 81, 82]. Mortality at 48 h and 14 days will be coded as a binary variable. Absolute and relative risk reduction will be reported with binomial 95% confidence intervals. Analysis will be by chi-squared test or Fisher exact test if the expected number of deaths is less than five in one or both treatment groups. In addition, we will present Kaplan-Meier survival curves comparing patients treated with iNO compared to placebo.

Time to death will be analysed using survival analysis (Cox proportional hazard model for difference between treatment arms).

Time to recovery. Among survivors (subgroup analysis of randomized participants), recovery times will be analysed by survival analysis (Cox proportional hazard model for difference between treatment arms). Time to sit unsupported, time to coma resolution (in the subset of patients with coma at study admission) and time to discharge will be documented by treating clinicians (blinded to intervention). Time to fever resolution, defined as the time required to achieve a temperature <38°C and the time to maintenance of temperature <38°C, will be determined from frequent vital sign monitoring. The time required to achieve a reduction in parasite density of 50%, 90% and to undetectable levels will be determined from daily blood smears. Results will be expressed as the median time to each event, with 95% confidence intervals.

Additional biomarkers (continuous variables, multiple measurements longitudinally collected) will be analysed using mixed-effects linear models, with the raw value or log-transformed value of the biomarker level as the dependent variable, as appropriate.

Neurocognitive outcomes. Statistical analysis of neurocognitive outcomes will be performed as follows. Frequencies of overall cognitive and neurologic deficits in children treated with iNO and children receiving placebo will be compared by X^2 test or Fisher's exact test. Differences in cognitive areas affected in the two age groups (18 mo-4 years, 5-10 years) will be assessed by comparing frequency of individuals with deficits in each area by X^2 test or Fisher's exact test. This will also serve as the best surrogate of level of impairment in a particular area in one age group vs another, since the type of testing for each cognitive area will be different for the two age groups, so no direct comparison of level of impairment will be possible across age groups. For individual tests, age-adjusted z-scores (determined from normative data in previous studies among healthy community controls) will be analyzed by means of mixed-effects models to examine study group differences in relation to neurocognitive outcomes. The models will provide estimated mean differences between children treated with iNO to placebo controls.

Interim analysis

An interim analysis for efficacy, safety and trial quality indices is planned at the midpoint of patient enrolment (approximately 45 patients per group). There is no plan to stop the trial prematurely for efficacy or futility based on the primary or secondary trial endpoints. Data at the time of the interim analysis will be presented to the Data Safety and Monitoring Board (DSMB) for review, who may advise that the trial continue without

modification, continue with changes to the protocol, or be discontinued prematurely. With respect to the statistical interpretation of safety data, the DSMB may recommend termination or modification of the trial if mortality rates exceed statistical thresholds as described in Appendix 1. However, we do not propose that the DSMB be strictly bound by pre-specified criteria, because of the complexity of the trade-offs between safety, efficacy, and the possibility that new information will change considerations. Rather, consideration of stopping guidelines requires a reasoned judgment based on all information that is available at the time of data review.

9.2 Number of subjects planned to be enrolled

We will enrol 180 patients (approximately 90 in each treatment arm).

Based on a recent clinical trial of L-arginine as adjunctive treatment for severe malaria, among patients who subsequently recovered, Ang-2 decreased by 2700pg/mL/day (95%CI 1800-3600pg/mL/day)[33]. We assume that a 50% change in this parameter would represent a clinically significant therapeutic effect. By standard calculations for normally distributed data, 80 patients per group will provide 80% power to detect a difference between two treatment arms of 1350pg/mL/day at p=0.05 (two-sided). This corresponds to a 50% difference in the rate of change of Ang-2 between the arms. To account for possible dropout, loss to follow-up, and/or non-evaluable data of 10% of patients, approximately 90 patients per study arm are required.

However, these calculations do not reflect the analytic plan. Therefore, a simulation study was performed. A number of assumptions needed to be made, such as within patient correlation and Ang-2 variability. Patient Ang-2 data were simulated using a

multivariate normal distribution. A simple autoregressive correlation structure was used with correlations of 0.75, 0.5 and 0.25 for lags 1, 2 and 3 respectively. In previous studies, variability appears proportional to the mean[33]. Simulations were run with three different relationships where the standard deviation was taken to be 40%, 50% and 80% of the Ang-2 mean at each time point. Both groups were assumed to start at Ang-2 levels of 15,000 pg/mL and the average values at each of days 1, 2 and 3 based on the hypothesized slopes of Ang-2. One thousand replications were performed at each standard deviation relationship and treatment effect. The mixed effects models were fit and the likelihood ratio test was used to test the hypothesis of no time by treatment interaction at the 5% level (two tailed). Table 4 shows the results of the power simulations.

Table 4: Power simulations for 90 patients per group

**Standard Deviation (% of mean)**	**Slope Difference**		
	**30% change (810 pg/mL/day)**	**40% change (1080 pg/mL/day)**	**50% change (1350 pg/mL/day)**
40	82%	97%	99%
50	66%	89%	98%
80	42%	60%	80%

Estimates from the Jinja Regional Hospital indicate that a minimum of 175 patients with moderately severe and severe malaria (excluding patients with SMA) are admitted annually. This implies that accrual of all patients to meet the required sample size will take approximately 12-18 months (180 patients with severe malaria).

At the interim analysis (midpoint of enrolment), we may perform a sample size re-calculation using the observed variance estimates, the observed means at each time in the placebo group, and the observed dropout rate (*a priori* estimate: 10%). Sample size may be increased if feasible and if suggested by this analysis at midpoint of enrolment.

9.3 Level of significance to be used

The threshold for statistical significance will be $\alpha=0.05$.

9.4 Criteria for termination of the trial

The trial will be terminated after enrolment of at least 90 patients in each arm, according to the *a priori* sample size calculation. As described above, the sample size may be re-calculated at the interim analysis, in which case the minimum number of patients per group may be increased accordingly.

The trial will also be continuously monitored for safety by an independent Data Safety and Monitoring Board (DSMB), who may recommend termination of the trial at an earlier point if unexpected concerns over patient safety arise.

9.5 Procedure for accounting for missing, unused, and spurious data

Primary endpoint. The primary endpoint of this trial involves collection of four serial (daily) venous blood samples for the measurement of rate of change of Ang-2. Missing data may therefore arise for a number of reasons including: premature discontinuation of study gas (not tolerated, safety issues, caregiver or health provider discretion); mortality event prior to complete collection of samples; or patient dropout. Our primary analysis using a mixed-effects linear model takes into account all available data points, and is able to handle missing data.

Secondary endpoints. Mortality at 48 hours is expected to be captured relatively completely, whereas 14-day mortality may be subject to loss to follow-up. Patients with missing data will not be included in computation of mortality, and we will report the number of missing cases. In the event that a significant mortality benefit is observed, we will perform a sensitivity analysis wherein patients with missing data are assigned an outcome of death (iNO group) or alive (placebo group), in order to systematically bias results toward the placebo group. If significant differences remain in this analysis, the result will be considered robust to adjustment for missing data. Our alternative analysis using Kaplan-Meier survival curves will censor patients at the point of last contact, in the case of missing data.

For the time to recovery endpoints, missing data will be handled by censoring patients at the time of the last documented time point. For other continuous variables, missing cases will be excluded from the analysis (two-way repeated-measures ANOVA), or will not affect the analysis (mixed-effects linear model).

9.6 Procedures for reporting deviations from the original statistical plan

Deviations from the *a priori* statistical analyses described in the protocol will be described and justified in the final report and will be reported as *post hoc* exploratory analyses.

9.7 Selection of subjects to be included in analyses

All randomized subjects will be included in the primary *intention-to-treat* analysis (change of Ang-2 over time). A secondary *per-protocol* analysis may be performed

including only patients with evaluable data who received the study gas for the proscribed duration (72 hours).

HIV co-infection, co-existent bacteremia and/or meningitis, and blood transfusion may affect Ang-2 levels and may represent effect modifiers of iNO treatment. We expect that randomization and blinding will ensure relatively even distribution of these co-variates between treatment and control groups, although these co-variates may add unexplained variance (random error) in the dependent variable (Ang-2). We will therefore conduct a subgroup analysis of iNO effect among HIV negative patients without bacteremia who do not undergo blood transfusion, to examine the role of iNO in this carefully selected patient population. Additional subgroup analyses will be conducted according to other possibly important co-variates: age < 5 years or ≥5 years; cerebral malaria or other severe malaria syndrome (respiratory distress); HIV seropositivity; and severe anemia.

10 DIRECT ACCESS TO SOURCE DATA AND DOCUMENTS

The investigators and institution (Department of Paediatrics, Jinja Hospital) will facilitate all trial-related monitoring, audits by institutional review boards (e.g., MUREC), and regulatory inspections by providing direct access to source data and documents.

11 ETHICS

Declaration of Helsinki

This study will be conducted at an international site (Uganda). Thus, the investigators will ensure that this study is conducted in full conformity with the Declaration of Helsinki

(1986), or with the ICH GCP regulations and guidelines, whichever affords greater protection to the trial participants.

Institutional Review Board

The Makerere University (Faculty of Medicine) Research and Ethics Committee (MUREC), together with the Institutional Review Board (IRB) of the University of Toronto will review this protocol. Both review boards will approve the study as well as the English and translated versions of the consent forms.

No deviation from, or changes of the protocol will be made without prior review and documented approval/favorable opinion from the IRB/independent or institutional ethics committee (IEC) of an amendment, except where necessary to eliminate an immediate hazard(s) to participants, or when the change(s) involve(s) only logistical or administrative aspects of the study (e.g., change of monitor(s), change of telephone number[s]). The investigator, or person designated by the investigator, will document and explain any deviation from the approved protocol. If the investigator implements a deviation from, or a change in, the protocol to eliminate an immediate hazard(s) to trial subjects without prior IRB/IEC approval/favorable opinion, the investigator will immediately submit the implemented deviation or change, the reasons for it, and, if appropriate, the proposed protocol amendment(s) to:

a) the Makerere University (Faculty of Medicine) Research and Ethics Committee (MUREC) for review and approval; and

b) Toronto Academic Health Sciences Network (TAHSN)

Informed Consent Process

Informed consent will be obtained from the parent/guardian of all children that will participate in the study. In addition, assent will be obtained from children eight years and older. The consent process shall be initiated at the time of enrolment into the study and shall continue throughout the child's participation. Parents or guardians of the children meeting the eligibility criteria for the study will have the study explained to them by one member of the study team. The consent will be done in the language that the parent/guardian best understands. If the parent/guardian provides consent for study participation, the parent/guardian will be given the consent forms to sign. A copy of the consent form will be given to the parent/guardian to keep while a duplicate copy will be kept in the patient's file. For children 8 years and older, in addition to the consent forms, the children will also have the study explained to them and asked to sign an assent form if they agree to participate in the study. For these older children, only those whose parent/guardian gives consent followed by the child giving assent will be enrolled in the study.

For illiterate caregivers, an independent witness will be present during the informed consent process and will sign the consent form as a witness. The caregivers may withdraw consent at any time throughout the course of the study, and this will be made clear in the informed consent process. A copy of the informed consent document will be given to the caregiver for their records. All individuals will be informed that there is no requirement to join the study and that standard medical care at Jinja Hospital will remain the same regardless of study enrolment.

If the parent/guardian chooses not to have their child participate in the study, the case will be turned over to the attending physician currently on duty at Jinja Hospital, Children's Unit, for routine care of their condition.

The present study will have 2 separate consent forms. The first consent form will be for participation in the study. This consent form gives permission to have blood samples taken. The second consent form will be to give permission for genetic studies on blood samples.

The study team based in the ER will be trained about the aims of the study and in how to appropriately seek consent or assent from the different individuals. They will then seek to obtain informed consent, which will be documented on a written compound or individual consent form.

Assent

Children 8 years and over are able to understand some of the concepts of our study testing and assent of these individuals will be sought prior to their enrolment in the study. These children will sign separate assent forms. The assent forms are short and simple and written in a way that can easily be explained and understood by a child of that age. We will also provide simplified information sheets for children 8 to 10 years of age.

Subject Confidentiality

Study participant information will be kept strictly confidential. Study data will be accessible only to study personnel. Study data will be stored long-term in files and computer databases in locked offices. Access to databases will be password-restricted,

and network security measures will be in place to ensure that information cannot be retrieved by personnel not involved with the study.

Study participant confidentiality is strictly held in trust by the participating investigators, their staff and the sponsors and their agents. This confidentiality includes testing of biological samples and genetic tests in addition to clinical information.

No information concerning the study or the data will be released to any unauthorized third party without prior approval of the sponsor. The study monitor or other authorized representatives may inspect all documents and records required to be maintained by the PI, and the study site will permit access to such records.

Investigation of critical illness pathways, including genetic determinants

Our present study will investigate whether iNO accelerates the restoration of endothelial quiescence during treatment for malaria. Because this study will prospectively collect samples from a well-defined cohort of children, we intend to investigate biomarkers and genetic factors that contribute to malaria pathogenesis and the response to iNO. The consent forms note that samples may be used to analyse biomarkers and genetic determinants of malaria pathogenesis, including pathways of endothelial activation, inflammation and coagulation. Individuals have the option on the consent form to refuse genetic testing on blood samples collected.

Following an approved and signed Materials Transfer Agreement (MTA), specimens will be maintained at the University of Toronto at the PI's laboratory.

Specimens will be numbered, so that specimens cannot be linked to a particular individual. Linkage to individuals' data information will be available only within the main database, to which non-study personnel will not have access and to which laboratory personnel will not have access at the time of any laboratory testing.

Community engagement

International collaborative research may face complex community challenges [91, 92]. Community engagement (CE) [93], a participatory process of collaboration and exchange between the various key stakeholders in the research process, may mitigate risks with respect to trial success, optimize participant retention, and minimize social disruption by providing a platform to seek input from and provide ongoing feedback to community members. There is currently no consensus on what CE activities are required in clinical trials, but a recently published model provides a useful framework of key CE activities and their ethical implications [3]. We propose to incorporate community engagement activities as part of our clinical trial, and have enlisted the academic guidance and expertise of Dr. James Lavery and colleagues, who operate one of the world's largest community engagement research programs, based at St. Michael's Hospital and the McLaughlin-Rotman Centre for Global Health.

These activities will seek to improve awareness of our trial and its findings in the catchment area of the Jinja Regional Referral Hospital. We also hope the CE activities will contribute to key ethical objectives for the trial, including respect for communities, fairness, transparency and accountability of the trial overall.

Key aims of the community outreach will include: (1) understanding the relevant community by consciously reaching out beyond the hospital to its catchment areas and listening to their issues and concerns; (2) providing information about the trial, including the pre-clinical evidence behind iNO, timeframe, procedures, and what will happen if the trial is successful; (3) building relationships and trust with local frontline healthcare workers; (4) specific educational/training activities, based on consultation with the nurses and/or frontline healthcare workers to ascertain what would be most relevant and beneficial for them; and (5) feedback of trial results, guided by the community itself as to how and what types of feedback activities would be most appropriate. Several levels of community will be targeted, including parents and primary caregivers of children, who comprise the group at highest risk of malaria, as well as healthcare professionals within the hospital catchment area.

These activities do not constitute a mechanism for recruitment of participants to the trial, since only children with severe malaria will be eligible. Instead the community engagement process is intended to build trust and avoid misunderstandings through a dynamic exchange of information and ideas between trial scientists and community members.

12 QUALITY CONTROL AND QUALITY ASSURANCE

Overview

Quality management (QM), both quality control and assurance, is a continuous, on-going process of evaluation of the quality of the conduct and documentation of studies. The QM process will be conducted in accordance with International Conference on

Harmonisation (ICH) guideline E6: Good Clinical Practice (GCP): Consolidated Guideline. Local quality control will be the responsibility of the Canadian and Ugandan PIs, on-site Pediatrician co-investigator, the trial manager, and study site coordinator.

The first step in QM will be training/re-training of the research staff to ensure consistency in data collection. Manuals of SOPs will be developed for all study related procedures and protocols. All staff will be trained in the SOPs and tested on knowledge of SOPs in written and practical tests. Retraining will occur for any project staff that do not know, understand or follow SOPs adequately. The next step for QM will be monitoring of collected data on a prospective basis. The site coordinator and/or trial manager will monitor collected data on a daily basis. The source documents shall be reviewed for completeness, accuracy and consistency. The last step in QM will be review of data entry. The site coordinator and/or trial manager will conduct data reviews weekly to ensure that data have been recorded and entered correctly. The weekly review will also determine if the study is being conducted in accordance with procedures identified in the protocol. Discrepancies in data entry will be recorded and the resolution of the discrepancy will be noted. These discrepancies will be reviewed with the nurses and medical officers weekly to discover the reason for errors, appropriately correct the errors, and ensure that the reasons for any ongoing or systematic errors are addressed in such a way that future errors are prevented.

A manual of SOPs for QM will also be developed and will be used to ensure that all aspects of the study meet the QM standards. This includes procedures to ensure that

data are being gathered and maintained in compliance with the study protocol and procedures to ensure that data are accurate.

Monitoring of ethical standards

In addition to supervision of data entry accuracy and adherence to protocol guidelines, the study site coordinator will be responsible for making sure that all study individuals complete appropriate training in research ethics and maintain up to date training in this area. In addition, any concerns about violations of ethical standards from the community or the team will be brought to the site PIs, who will record the specific allegation, investigate the allegation, and discuss the findings with the PIs. The PIs will complete the investigation, address the concern, and record the investigation and outcome in study records.

Laboratory analyses, sample collection and storage

The study site coordinator and/or trial manager will manage the study sample handling and storage. The site coordinator, trial manager and/or Ugandan PI will review sample handling/storage/location/shipping with the CoreLab supervisor from CoreLab monthly. Discrepancies will be noted, recorded, discussed and resolved with the Ugandan PI.

A manual of SOPs will be developed to specify how all the laboratory samples shall be collected, transported, tested and reported.

Protocol Deviations

Periodic review of how the protocol is being followed has been discussed above. Minor variations should be captured by QC of the consents and questionnaires, and these will be addressed on a prospective, corrective-action approach with logging in of incident

reports. The sum of these problems and corrective actions will be reviewed regularly by the trial manager and the PI to ensure reliability of the study. In addition, minor protocol deviations such as minor changes in methods of collection, forms, laboratory tests, etc, which occur in every scientific study as part of the process of optimizing the study, will be recorded and passed on in the reports to the governing IRBs. The same will apply to changes that may be required such as changing of collection timing due to weather, transmission, power availability, problems with laboratory testing, etc; enrollment of additional individuals if follow-up loss is larger than anticipated, and other such issues that are likely to come up and that do not constitute significant conceptual, ethical or scientific protocol deviation. Planned major deviations from the protocol (eg, increasing frequency of testing, changing study site) will require consultation with and approval from the governing IRBs.

A protocol deviation is any noncompliance with the clinical trial protocol or GCP requirements. The noncompliance may be either on the part of the subject, the investigator, or the study site staff. As a result of deviations, corrective actions are to be developed by the site and implemented promptly.

These practices are consistent with ICH E6:

4.5 Compliance with Protocol, sections 4.5.1, 4.5.2, and 4.5.3

5.1 Quality Assurance and Quality Control, section 5.1.1

5.20 Noncompliance, sections 5.20.1, and 5.20.2.

All deviations from the protocol must be addressed in study subject source documents. A completed study Protocol Deviation Form will be maintained in the regulatory file, as well as in the subject's source document. Protocol deviations must be sent to the ethics review boards (MUREC and University of Toronto) as per their guidelines. The site PI and trial manager are responsible for knowing and adhering to their IRB/IEC requirements.

Role of the DSMB

A DSMB will provide independent oversight of the trial quality (as well as safety and efficacy monitoring). Quarterly reports will be submitted to the DSMB chairperson summarizing progress of patient accrual in the trial, and indicators of trial quality (proportion of patients receiving the intervention according to protocol, completeness of data). The DSMB will meet as a group at the midpoint of trial enrolment to review data related to trial quality. At this point, the DSMB may recommend the trial proceed without modifications, proceed with modifications to the protocol, or be terminated.

Audit

A trial monitor will audit the trial documentation prior to its initiation, and at the midpoint of enrolment. Feedback will be used to modify the protocol and/or SOPs.

13 DATA HANDLING AND RECORDKEEPING

Data Management Responsibilities

The medical officers in ACU will complete the study forms detailing the clinical data of the patients. The nurses will also complete observation charts about the patients' clinical condition that will be part of the clinical data. These forms will be collected and will form the clinical data for each study participant's folder. A folder will be created as

soon as a patient is enrolled into the study. These folders will be kept in the data room in a locked cabinet. At admission, the medical history and physical examination forms will be completed. All data collection will be performed according to the SOPs for each type of testing, which will contain complete information for filling out each form, data entry, cross-checking of data entry, data cleaning, storage and back-up.

Data Storage and Back-up

Hard copies of forms will be kept in the study data room as described above. Final database files will be also be kept in the data room in the CM project offices in MU-UMN computers and on a back-up drive. Daily back-up of database files to a back-up drive will be performed as data is entered. Files will be transferred to/from the University of Toronto by secure password-protected documents.

Timing/Reports

Data review will be an ongoing process. Systematic data analysis, review and summaries will be performed regularly as outlined in the data reports SOP. Reports will be produced annually unless there is need to report an unexpected result or problem after the findings has been confirmed and analyzed.

Data analysis will be also be ongoing, to allow identification of problems with data, data collection or type of analysis. Preliminary findings that demonstrate significant results may be presented as abstracts or talks. Final publications will be based on the originally planned data analysis, or on appropriate modifications of this plan, if it becomes clear that modifications or changes are necessary to best examine the data.

Study Records Retention

Paper copies of records will be maintained until the end of the study and for at least 10 years after that point, so that data can be cross-checked and verified with hard copies if necessary. After 10 years, if electronic records are determined to be accurate, hard copies will be destroyed. Electronic records will be maintained on password-protected computers and storage drives indefinitely. No special permission is required prior to destruction of records.

14 PUBLICATION POLICY

A clear set of guidelines, outlined below, will be given to all potential authors of papers generated by this study. First authors will be expected to have been a major/the major contributor to the work outlined in the paper and to have written the first draft of the paper. As outlined below, all authors must have significantly contributed to the work outlined in the paper. The PI will make the final decisions on authorship for papers resulting from this study.

1. Individuals will be considered for inclusion as authors on work submitted for publications if they have provided:

- significant contributions affecting the direction, scope or depth of research
- long-term guidance and development of the project
- creative contributions to the project with clear understanding of its goals
- development of methodologies necessary for timely completion of the project
- data analysis of interpretation vital to conclusions of the project

2. Individuals will not be included as authors for contributions strictly limited to:

- providing laboratory space or use of instrumentation
- providing funding
- services, consulting, or materials provided for a fee or reimbursement
- involvement in patient care or providing patient samples
- routine technical work (as provided by any individual in the lab)
- status as a supervisor, section head, department chairperson
- proofreading or editing of manuscripts
- advice given to solve problems that are narrowly defined or unrelated to the project objective

3. Responsibilities

a) Primary author will:

- Inform all authors and contributors as to how their contributions will be acknowledged.
- Be able to identify the specific contribution of each author.
- Understand the general principles of all work included in a paper.
- Be willing to share openly the data obtained and methodology utilized in the investigation.

b) All authors:

- Be able to defend the methodology and data pertinent to their specific contributions to the project.
- Agree with the general conclusions and interpretations of the paper.

4. Content

- All manuscripts should serve to represent an accurate and complete reflection of the methods utilized and the data obtained in the investigative effort.
- In a publication, all data pertinent to the project should be reported, whether supportive or unsupportive of the thesis or conclusions.
- Except for review articles, publishing the same material in more than 1 paper should be avoided.
- Unnecessary fragmentation of a complete body of work into separate publications should be avoided.
- When ideas, concepts, or text of others are used, appropriate citations should be made.
- Prior work in the field should be referenced appropriately.
- The source of funding should be identified when a work is published.

Following completion of the study, the investigator is expected to publish the results of this research in a scientific journal. The International Committee of Medical Journal Editors (ICMJE) member journals have adopted a trials-registration policy as a condition for publication. This policy requires that all clinical trials be registered in a public trials registry such as ClinicalTrials.gov[94], which is sponsored by the National Library of Medicine. Other biomedical journals are considering adopting similar policies. Any clinical trial starting enrollment after 01 July 2005 must be registered on or before patient enrollment. The ICMJE defines a clinical trial as any research project that prospectively assigns human subjects to intervention or comparison groups to study the cause-and-effect relationship between a medical intervention and a health outcome.

REFERENCES

1. Snow, R.W., et al., *The global distribution of clinical episodes of Plasmodium falciparum malaria.* Nature, 2005. **434**(7030): p. 214-7.
2. Newton, C.R. and S. Krishna, *Severe falciparum malaria in children: current understanding of pathophysiology and supportive treatment.* Pharmacol Ther, 1998. **79**(1): p. 1-53.
3. Gramaglia, I., et al., *Low nitric oxide bioavailability contributes to the genesis of experimental cerebral malaria.* Nat Med, 2006. **12**(12): p. 1417-22.
4. Yeo, T.W., et al., *Impaired nitric oxide bioavailability and L-arginine reversible endothelial dysfunction in adults with falciparum malaria.* J Exp Med, 2007. **204**(11): p. 2693-704.
5. Bogdan, C., *Nitric oxide and the immune response.* Nat Immunol, 2001. **2**(10): p. 907-16.
6. Korhonen, R., et al., *Nitric oxide production and signaling in inflammation.* Curr Drug Targets Inflamm Allergy, 2005. **4**(4): p. 471-9.
7. Baylis, C. and P. Vallance, *Measurement of nitrite and nitrate levels in plasma and urine--what does this measure tell us about the activity of the endogenous nitric oxide system?* Curr Opin Nephrol Hypertens, 1998. **7**(1): p. 59-62.
8. Moncada, S., R.M. Palmer, and E.A. Higgs, *Nitric oxide: physiology, pathophysiology, and pharmacology.* Pharmacol Rev, 1991. **43**(2): p. 109-42.
9. Gessler, P., et al., *A new side effect of inhaled nitric oxide in neonates and infants with pulmonary hypertension: functional impairment of the neutrophil respiratory burst.* Intensive Care Med, 1996. **22**(3): p. 252-8.
10. Kubes, P., M. Suzuki, and D.N. Granger, *Nitric oxide: an endogenous modulator of leukocyte adhesion.* Proc Natl Acad Sci U S A, 1991. **88**(11): p. 4651-5.
11. van der Veen, R.C., *Nitric oxide and T helper cell immunity.* Int Immunopharmacol, 2001. **1**(8): p. 1491-500.
12. Bryk, R., P. Griffin, and C. Nathan, *Peroxynitrite reductase activity of bacterial peroxiredoxins.* Nature, 2000. **407**(6801): p. 211-5.
13. Luckhart, S., et al., *The mosquito Anopheles stephensi limits malaria parasite development with inducible synthesis of nitric oxide.* Proc Natl Acad Sci U S A, 1998. **95**(10): p. 5700-5.
14. Ribeiro, J.M., et al., *Reversible binding of nitric oxide by a salivary heme protein from a bloodsucking insect.* Science, 1993. **260**(5107): p. 539-41.
15. Rockett, K.A., et al., *Killing of Plasmodium falciparum in vitro by nitric oxide derivatives.* Infect Immun, 1991. **59**(9): p. 3280-3.
16. Sobolewski, P., et al., *Nitric oxide bioavailability in malaria.* Trends Parasitol, 2005. **21**(9): p. 415-22.
17. Favre, N., B. Ryffel, and W. Rudin, *The development of murine cerebral malaria does not require nitric oxide production.* Parasitology, 1999. **118 (Pt 2)**: p. 135-8.
18. Kremsner, P.G., et al., *Malaria antigen and cytokine-induced production of reactive nitrogen intermediates by murine macrophages: no relevance to the development of experimental cerebral malaria.* Immunology, 1993. **78**(2): p. 286-90.
19. Favre, N., B. Ryffel, and W. Rudin, *Parasite killing in murine malaria does not require nitric oxide production.* Parasitology, 1999. **118 (Pt 2)**: p. 139-43.
20. van der Heyde, H.C., et al., *Nitric oxide is neither necessary nor sufficient for resolution of Plasmodium chabaudi malaria in mice.* J Immunol, 2000. **165**(6): p. 3317-23.

21. Jacobs, P., D. Radzioch, and M.M. Stevenson, *Nitric oxide expression in the spleen, but not in the liver, correlates with resistance to blood-stage malaria in mice.* J Immunol, 1995. **155**(11): p. 5306-13.
22. Gillman, B.M., et al., *Suppression of Plasmodium chabaudi parasitemia is independent of the action of reactive oxygen intermediates and/or nitric oxide.* Infect Immun, 2004. **72**(11): p. 6359-66.
23. Amante, F.H. and M.F. Good, *Prolonged Th1-like response generated by a Plasmodium yoelii-specific T cell clone allows complete clearance of infection in reconstituted mice.* Parasite Immunol, 1997. **19**(3): p. 111-26.
24. Hobbs, M.R., et al., *A new NOS2 promoter polymorphism associated with increased nitric oxide production and protection from severe malaria in Tanzanian and Kenyan children.* Lancet, 2002. **360**(9344): p. 1468-75.
25. Cramer, J.P., et al., *Age-dependent effect of plasma nitric oxide on parasite density in Ghanaian children with severe malaria.* Trop Med Int Health, 2005. **10**(7): p. 672-80.
26. Day, N.P., et al., *The pathophysiologic and prognostic significance of acidosis in severe adult malaria.* Crit Care Med, 2000. **28**(6): p. 1833-40.
27. MacPherson, G.G., et al., *Human cerebral malaria. A quantitative ultrastructural analysis of parasitized erythrocyte sequestration.* Am J Pathol, 1985. **119**(3): p. 385-401.
28. Turner, G.D., et al., *An immunohistochemical study of the pathology of fatal malaria. Evidence for widespread endothelial activation and a potential role for intercellular adhesion molecule-1 in cerebral sequestration.* Am J Pathol, 1994. **145**(5): p. 1057-69.
29. De Caterina, R., et al., *Nitric oxide decreases cytokine-induced endothelial activation. Nitric oxide selectively reduces endothelial expression of adhesion molecules and proinflammatory cytokines.* J Clin Invest, 1995. **96**(1): p. 60-8.
30. Jakobsen, P.H., et al., *Increased plasma concentrations of sICAM-1, sVCAM-1 and sELAM-1 in patients with Plasmodium falciparum or P. vivax malaria and association with disease severity.* Immunology, 1994. **83**(4): p. 665-9.
31. de Mast, Q., et al., *Thrombocytopenia and release of activated von Willebrand Factor during early Plasmodium falciparum malaria.* J Infect Dis, 2007. **196**(4): p. 622-8.
32. Hollestelle, M.J., et al., *von Willebrand factor propeptide in malaria: evidence of acute endothelial cell activation.* Br J Haematol, 2006. **133**(5): p. 562-9.
33. Yeo, T.W., et al., *Angiopoietin-2 is associated with decreased endothelial nitric oxide and poor clinical outcome in severe falciparum malaria.* Proc Natl Acad Sci U S A, 2008. **105**(44): p. 17097-102.
34. Lovegrove, F.E., et al., *Serum angiopoietin-1 and -2 levels discriminate cerebral malaria from uncomplicated malaria and predict clinical outcome in African children.* PLoS One, 2009. **4**(3): p. e4912.
35. Fiedler, U., et al., *Angiopoietin-2 sensitizes endothelial cells to TNF-alpha and has a crucial role in the induction of inflammation.* Nat Med, 2006. **12**(2): p. 235-9.
36. Casals-Pascual, C., et al., *High levels of erythropoietin are associated with protection against neurological sequelae in African children with cerebral malaria.* Proc Natl Acad Sci U S A, 2008. **105**(7): p. 2634-9.
37. Matsushita, K., et al., *Nitric oxide regulates exocytosis by S-nitrosylation of N-ethylmaleimide-sensitive factor.* Cell, 2003. **115**(2): p. 139-50.
38. Deanfield, J.E., J.P. Halcox, and T.J. Rabelink, *Endothelial function and dysfunction: testing and clinical relevance.* Circulation, 2007. **115**(10): p. 1285-95.
39. Marsh, K., et al., *Indicators of life-threatening malaria in African children.* N Engl J Med, 1995. **332**(21): p. 1399-404.

40. Mohan, A., S.K. Sharma, and S. Bollineni, *Acute lung injury and acute respiratory distress syndrome in malaria.* J Vector Borne Dis, 2008. **45**(3): p. 179-93.
41. Duarte, M.I., et al., *Ultrastructure of the lung in falciparum malaria.* Am J Trop Med Hyg, 1985. **34**(1): p. 31-5.
42. Lovegrove, F.E., et al., *Parasite burden and CD36-mediated sequestration are determinants of acute lung injury in an experimental malaria model.* PLoS Pathog, 2008. **4**(5): p. e1000068.
43. Speyer, C.L., et al., *Regulatory effects of iNOS on acute lung inflammatory responses in mice.* Am J Pathol, 2003. **163**(6): p. 2319-28.
44. Zeidler, P.C., L.M. Millecchia, and V. Castranova, *Role of inducible nitric oxide synthase-derived nitric oxide in lipopolysaccharide plus interferon-gamma-induced pulmonary inflammation.* Toxicol Appl Pharmacol, 2004. **195**(1): p. 45-54.
45. Benzing, A. and K. Geiger, *Inhaled nitric oxide lowers pulmonary capillary pressure and changes longitudinal distribution of pulmonary vascular resistance in patients with acute lung injury.* Acta Anaesthesiol Scand, 1994. **38**(7): p. 640-5.
46. Benzing, A., et al., *Inhaled nitric oxide reduces pulmonary transvascular albumin flux in patients with acute lung injury.* Anesthesiology, 1995. **83**(6): p. 1153-61.
47. Razavi, H.M., et al., *Pulmonary neutrophil infiltration in murine sepsis: role of inducible nitric oxide synthase.* Am J Respir Crit Care Med, 2004. **170**(3): p. 227-33.
48. Sato, Y., et al., *Nitric oxide reduces the sequestration of polymorphonuclear leukocytes in lung by changing deformability and CD18 expression.* Am J Respir Crit Care Med, 1999. **159**(5 Pt 1): p. 1469-76.
49. Neviere, R., et al., *Inhaled nitric oxide modulates leukocyte kinetics in the mesenteric venules of endotoxemic rats.* Crit Care Med, 2000. **28**(4): p. 1072-6.
50. Kun, J.F., et al., *Nitric oxide synthase 2(Lambarene) (G-954C), increased nitric oxide production, and protection against malaria.* J Infect Dis, 2001. **184**(3): p. 330-6.
51. Dhangadamajhi, G., et al., *The CCTTT pentanucleotide microsatellite in iNOS promoter influences the clinical outcome in P. falciparum infection.* Parasitol Res, 2009. **104**(6): p. 1315-20.
52. Boutlis, C.S., et al., *Inducible nitric oxide synthase (NOS2) promoter CCTTT repeat polymorphism: relationship to in vivo nitric oxide production/NOS activity in an asymptomatic malaria-endemic population.* Am J Trop Med Hyg, 2003. **69**(6): p. 569-73.
53. Ohashi, J., et al., *Significant association of longer forms of CCTTT Microsatellite repeat in the inducible nitric oxide synthase promoter with severe malaria in Thailand.* J Infect Dis, 2002. **186**(4): p. 578-81.
54. Kun, J.F., et al., *Polymorphism in promoter region of inducible nitric oxide synthase gene and protection against malaria.* Lancet, 1998. **351**(9098): p. 265-6.
55. Cramer, J.P., et al., *iNOS promoter variants and severe malaria in Ghanaian children.* Trop Med Int Health, 2004. **9**(10): p. 1074-80.
56. Burgner, D., et al., *Inducible nitric oxide synthase polymorphism and fatal cerebral malaria.* Lancet, 1998. **352**(9135): p. 1193-4.
57. Dhangadamajhi, G., et al., *Endothelial nitric oxide synthase gene polymorphisms and Plasmodium falciparum infection in Indian adults.* Infect Immun, 2009. **77**(7): p. 2943-7.
58. Dhangadamajhi, G., et al., *Genetic variation in neuronal nitric oxide synthase (nNOS) gene and susceptibility to cerebral malaria in Indian adults.* Infect Genet Evol, 2009. **9**(5): p. 908-11.
59. Anstey, N.M., et al., *Nitric oxide in Tanzanian children with malaria: inverse relationship between malaria severity and nitric oxide production/nitric oxide synthase type 2 expression.* J Exp Med, 1996. **184**(2): p. 557-67.

60. Lopansri, B.K., et al., *Low plasma arginine concentrations in children with cerebral malaria and decreased nitric oxide production.* Lancet, 2003. **361**(9358): p. 676-8.
61. Aslan, M., et al., *Oxygen radical inhibition of nitric oxide-dependent vascular function in sickle cell disease.* Proc Natl Acad Sci U S A, 2001. **98**(26): p. 15215-20.
62. Thumwood, C.M., et al., *Antioxidants can prevent cerebral malaria in Plasmodium berghei-infected mice.* Br J Exp Pathol, 1989. **70**(3): p. 293-303.
63. Cosby, K., et al., *Nitrite reduction to nitric oxide by deoxyhemoglobin vasodilates the human circulation.* Nat Med, 2003. **9**(12): p. 1498-505.
64. Dondorp, A., et al., *Artesunate versus quinine for treatment of severe falciparum malaria: a randomised trial.* Lancet, 2005. **366**(9487): p. 717-25.
65. Serghides, L., et al., *Rosiglitazone modulates the innate immune response to Plasmodium falciparum infection and improves outcome in experimental cerebral malaria.* J Infect Dis, 2009. **199**(10): p. 1536-45.
66. Finer, N.N. and K.J. Barrington, *Nitric oxide for respiratory failure in infants born at or near term.* Cochrane Database Syst Rev, 2006(4): p. CD000399.
67. Sokol, J., S.E. Jacobs, and D. Bohn, *Inhaled nitric oxide for acute hypoxemic respiratory failure in children and adults.* Cochrane Database Syst Rev, 2003(1): p. CD002787.
68. Barrington, K.J. and N.N. Finer, *Inhaled nitric oxide for respiratory failure in preterm infants.* Cochrane Database Syst Rev, 2007(3): p. CD000509.
69. Adhikari, N.K., et al., *Effect of nitric oxide on oxygenation and mortality in acute lung injury: systematic review and meta-analysis.* BMJ, 2007. **334**(7597): p. 779.
70. *Inhaled nitric oxide in full-term and nearly full-term infants with hypoxic respiratory failure. The Neonatal Inhaled Nitric Oxide Study Group.* N Engl J Med, 1997. **336**(9): p. 597-604.
71. Davidson, D., et al., *Inhaled nitric oxide for the early treatment of persistent pulmonary hypertension of the term newborn: a randomized, double-masked, placebo-controlled, dose-response, multicenter study. The I-NO/PPHN Study Group.* Pediatrics, 1998. **101**(3 Pt 1): p. 325-34.
72. Dobyns, E.L., et al., *Multicenter randomized controlled trial of the effects of inhaled nitric oxide therapy on gas exchange in children with acute hypoxemic respiratory failure.* J Pediatr, 1999. **134**(4): p. 406-12.
73. Michael, J.R., et al., *Inhaled nitric oxide versus conventional therapy: effect on oxygenation in ARDS.* Am J Respir Crit Care Med, 1998. **157**(5 Pt 1): p. 1372-80.
74. Dellinger, R.P., et al., *Effects of inhaled nitric oxide in patients with acute respiratory distress syndrome: results of a randomized phase II trial. Inhaled Nitric Oxide in ARDS Study Group.* Crit Care Med, 1998. **26**(1): p. 15-23.
75. Troncy, E., et al., *Inhaled nitric oxide in acute respiratory distress syndrome: a pilot randomized controlled study.* Am J Respir Crit Care Med, 1998. **157**(5 Pt 1): p. 1483-8.
76. Lundin, S., et al., *Inhalation of nitric oxide in acute lung injury: results of a European multicentre study. The European Study Group of Inhaled Nitric Oxide.* Intensive Care Med, 1999. **25**(9): p. 911-9.
77. Gerlach, H., et al., *Dose-response characteristics during long-term inhalation of nitric oxide in patients with severe acute respiratory distress syndrome: a prospective, randomized, controlled study.* Am J Respir Crit Care Med, 2003. **167**(7): p. 1008-15.
78. Taylor, R.W., et al., *Low-dose inhaled nitric oxide in patients with acute lung injury: a randomized controlled trial.* JAMA, 2004. **291**(13): p. 1603-9.
79. Germann, P., et al., *Inhaled nitric oxide therapy in adults: European expert recommendations.* Intensive Care Med, 2005. **31**(8): p. 1029-41.

80. Idro, R., et al., *Severe malaria in children in areas with low, moderate and high transmission intensity in Uganda.* Trop Med Int Health, 2006. **11**(1): p. 115-24.
81. Tran, T.H., et al., *A controlled trial of artemether or quinine in Vietnamese adults with severe falciparum malaria.* N Engl J Med, 1996. **335**(2): p. 76-83.
82. van Hensbroek, M.B., et al., *A trial of artemether or quinine in children with cerebral malaria.* N Engl J Med, 1996. **335**(2): p. 69-75.
83. Newton, C.R., et al., *The prognostic value of measures of acid/base balance in pediatric falciparum malaria, compared with other clinical and laboratory parameters.* Clin Infect Dis, 2005. **41**(7): p. 948-57.
84. Waller, D., et al., *Clinical features and outcome of severe malaria in Gambian children.* Clin Infect Dis, 1995. **21**(3): p. 577-87.
85. Duke, T.D., W. Butt, and M. South, *Predictors of mortality and multiple organ failure in children with sepsis.* Intensive Care Med, 1997. **23**(6): p. 684-92.
86. Hindy-Francois, C., et al., *Admission base deficit as a long-term prognostic factor in severe pediatric trauma patients.* J Trauma, 2009. **67**(6): p. 1272-7.
87. Molina Hazan, V., et al., *Blood Lactate Levels Differ Significantly Between Surviving and Nonsurviving Patients Within the Same Risk-Adjusted Classification for Congenital Heart Surgery (RACHS-1) Group After Pediatric Cardiac Surgery.* Pediatr Cardiol.
88. Hrobjartsson, A., et al., *Blinded trials taken to the test: an analysis of randomized clinical trials that report tests for the success of blinding.* Int J Epidemiol, 2007. **36**(3): p. 654-63.
89. Clark, R.H., et al., *Low-dose nitric oxide therapy for persistent pulmonary hypertension of the newborn. Clinical Inhaled Nitric Oxide Research Group.* N Engl J Med, 2000. **342**(7): p. 469-74.
90. Kyu, H.H. and E. Fernandez, *Artemisinin derivatives versus quinine for cerebral malaria in African children: a systematic review.* Bull World Health Organ, 2009. **87**(12): p. 896-904.
91. Newman, P.A., *Towards a science of community engagement.* Lancet, 2006. **367**(9507): p. 302.
92. Tindana, P.O., et al., *Grand challenges in global health: community engagement in research in developing countries.* PLoS Med, 2007. **4**(9): p. e273.
93. Lavery, J.V., et al., *Towards a framework for community engagement in global health research.* Trends Parasitol. **26**(6): p. 279-83.
94. De Angelis, C., et al., *Clinical trial registration: a statement from the International Committee of Medical Journal Editors.* N Engl J Med, 2004. **351**(12): p. 1250-1.
95. Svolba, G. and P. Bauer, *Statistical quality control in clinical trials.* Control Clin Trials, 1999. **20**(6): p. 519-30.

APPENDIX 1. STATISTICAL THRESHOLDS FOR MONITORING MORTALITY

The frequency of deaths and severe adverse events in the trial will be carefully monitored for deviations from expected baseline level using statistical control charts. The control chart is a commonly used tool to monitor output of processes in a variety of settings, including clinical trials[95]. This method continuously follows a process outcome (e.g., patient mortality), allowing early detection of deviations from a state of "statistical control," thereby prompting a search for assignable causes.

For each consecutive group of 10 patients (irrespective of group assignment), the mortality will be computed and plotted on the control chart. Points lying above the upper control limit (UCL) will trigger trial halting and a review of trial procedures for possible causes of elevated mortality and initiating corrective measures. The probability of a type I error ("false alarm," halting the trial unnecessarily) and type II error (when the chart fails to indicate the presence of a deviation from baseline mortality) under this scheme can be quantified with some assumptions.

We determined an expected level of mortality from a literature review of clinical trials involving severe malaria in children in sub-Saharan Africa[90]. The mortality in these controlled trials ranged from 6-29%, with a median of 19%. Data from Uganda indicates that the mortality associated with severe malaria is comparable (16% of children with impaired consciousness, and 21% of children with deep acidotic breathing)[80]. The mortality rate observed in the clinical trial is therefore expected to be approximately

20%, with random variation about this baseline value as we sample small groups of patients for monitoring purposes.

The upper control limit was determined using a traditional approach under assumptions that the distribution of mortality will follow a normal approximation to the binomial distribution, and balancing the “detection capacity” (mimimizing type II error rate) while minimizing the probability of “false alarms” (type I error rate). This yielded an upper control limit of 57%, or a threshold of 6 deaths in a sample of 10 patients.

An index of the effectiveness of a process control method is the time needed to detect a change after it occurs. As long as a clinical trial runs along an expected level of mortality, the length of the run (the number of inspections for mortality) up to an “out-of-control alarm” should be large. Unnecessary correcting actions, which may be costly and time consuming, should be avoided. Using the process control scheme proposed, if mortality remains constant at 20%, the average run length (ARL) leading to a “false alarm” is 157 inspections (1570 patients), and in our trial of 180 patients, the probability of halting the trial unnecessarily for random variations in the mortality is at most 10.8%. On the other hand, if a significant change occurs in the process, the number of inspections needed to detect it should be as small as possible. This helps initiate actions to investigate the cause of the elevated mortality as quickly as possible. Under the proposed monitoring scheme, if the mortality rate doubles from 20% to 40%, an ARL of 6 inspections (60 patients) will lead to a “true alarm,” prompting halting of the trial and review of procedures for remediable actions.

In addition to the continuous monitoring of sequential groups of 10 patients, we will follow cumulative mortality as an additional safety measure. Mortality in each group will be plotted on (Figures 1 to 3), without unblinding. The unblinded trial manager will be responsible for generating this data after every 10 patients are enrolled. If the cumulative in either group exceeds the upper confidence limit, the trial will be halted and the DSMB will be notified. The DSMB will then be convened to review the safety data and decide if the trial should continue per protocol, proceed with caution, be further investigated, be discontinued or be modified and then proceed.

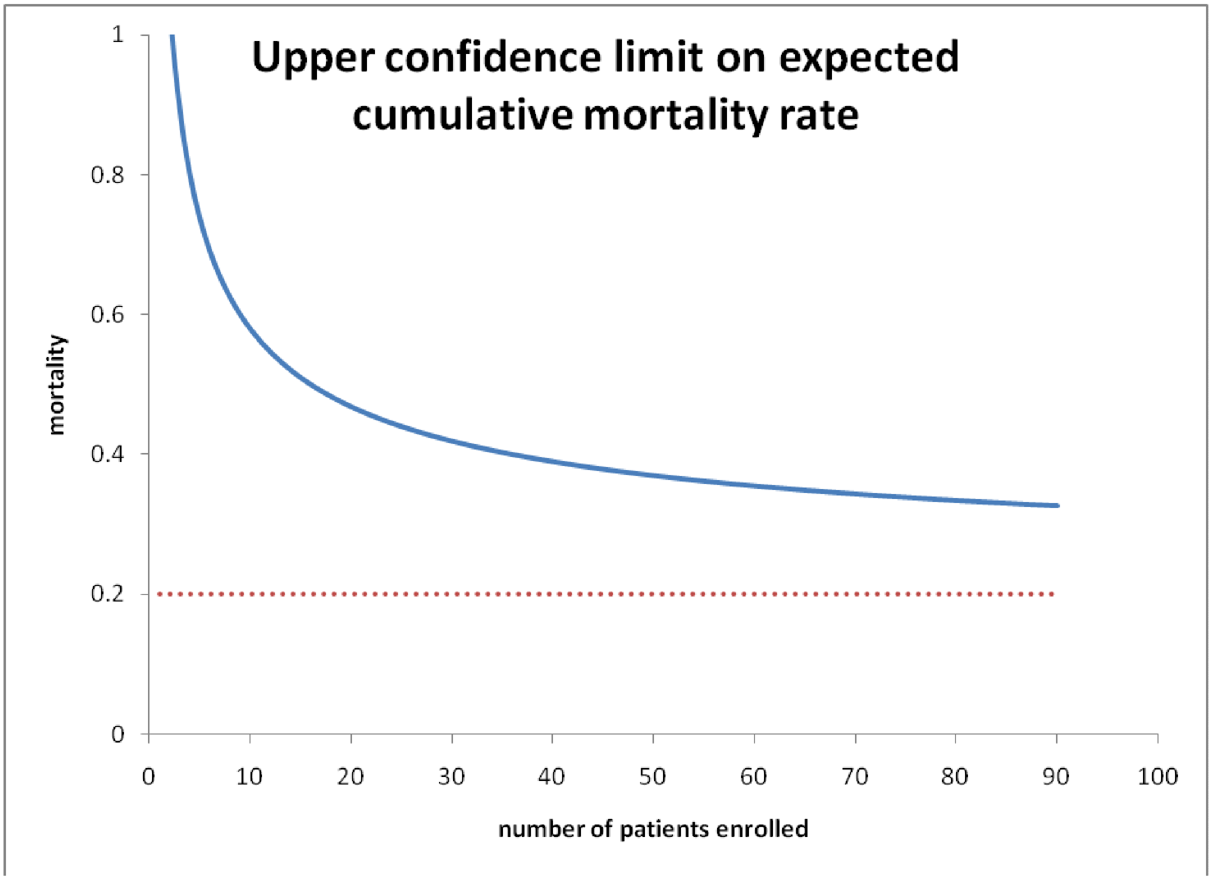

Figure 1. Expected mortality rate (dotted red line) and upper confidence limit (solid blue line). Plotting the observed mortality rate in real time throughout the trial will allow early detection of mortality beyond the expected rate (prompting trial halting for safety review).

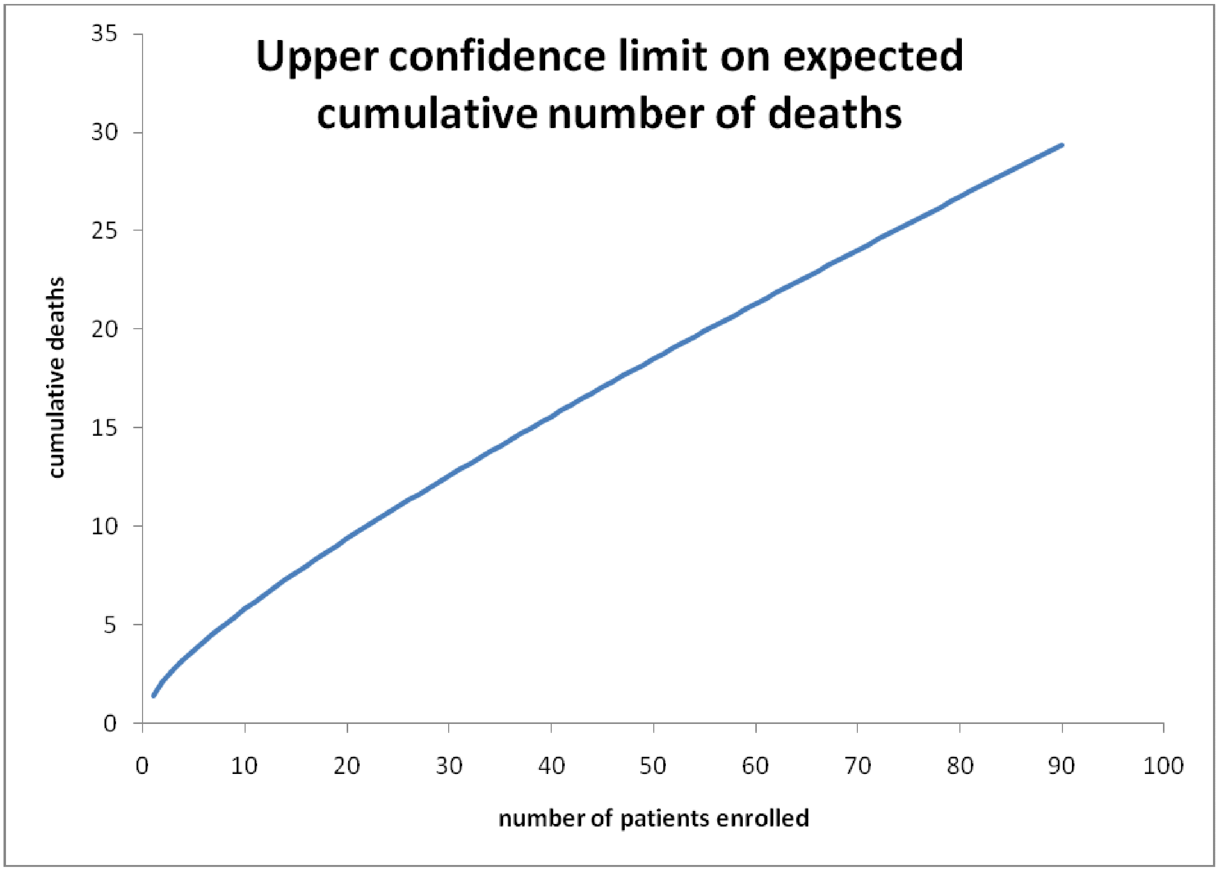

Figure 2. Upper confidence limit (solid blue line) on the expected cumulative number of deaths. Plotting the observed number of deaths in real time throughout the trial will allow early detection of mortality beyond the expected rate (prompting trial halting for safety review).

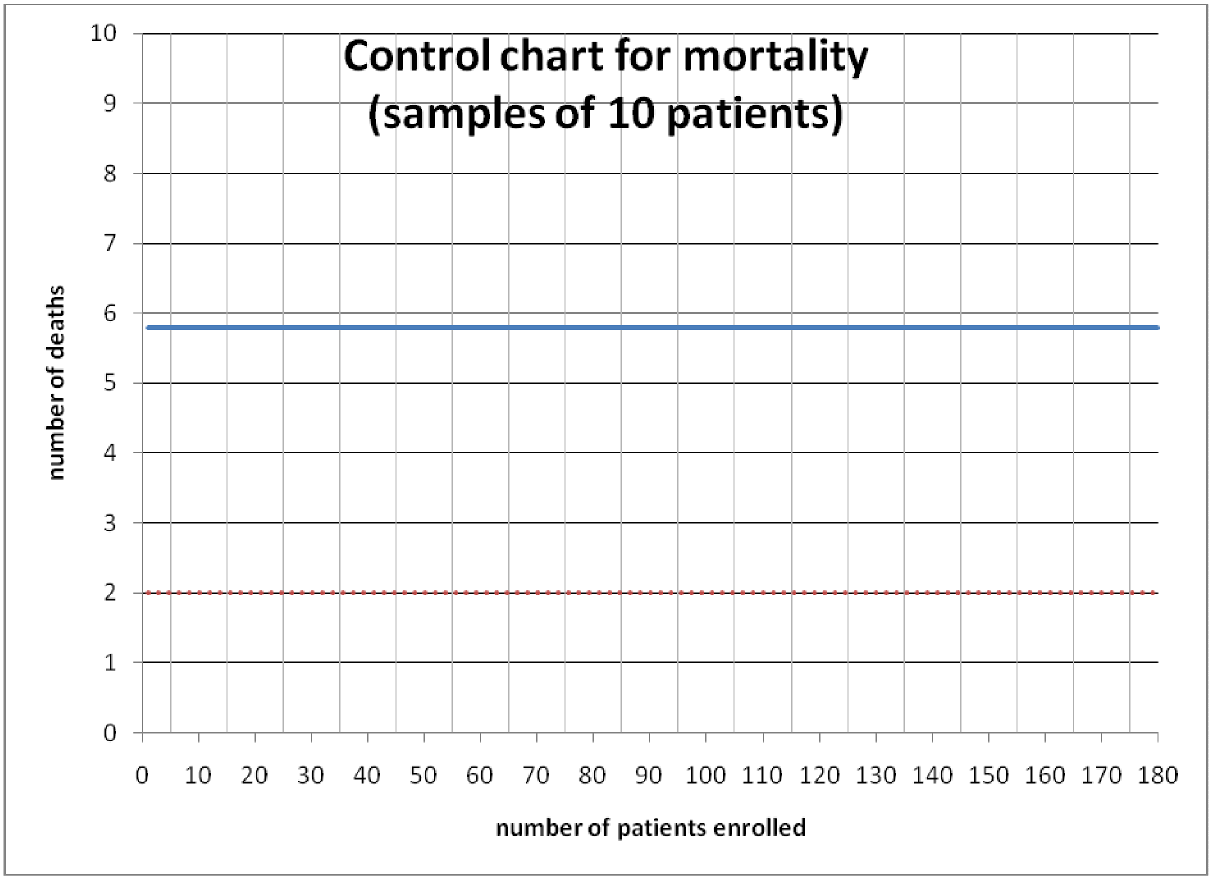

Figure 3. Control chart for continuous monitoring of mortality in consecutive samples of 10 patients enrolled in the trial. Expected mortality rate (dotted red line) and upper confidence limit (solid blue line) are shown. Plotting the observed number of deaths in consecutive samples of 10 patients in real time throughout the trial will allow early detection of mortality beyond the expected rate (prompting trial halting for safety review).
